# Structure of a 13-fold superhelix (almost) determined from first principles

**DOI:** 10.1107/S2052252515000238

**Published:** 2015-01-27

**Authors:** Guillaume A. Schoch, Massimo Sammito, Claudia Millán, Isabel Usón, Markus G. Rudolph

**Affiliations:** a Molecular Design and Chemical Biology, F. Hoffmann-La Roche Ltd, Grenzacherstrasse 124, 4070 Basel, Switzerland; b Instituto de Biología Molecular de Barcelona (IBMB), CSIC, Barcelona Science Park, Baldiri Reixach 15, 08028 Barcelona, Spain; c Institucio Catalana de Recerca i Estudis Avançats, Passeig Lluis Companys, 23, 08010 Barcelona, Spain

**Keywords:** glucocorticoid receptor co-activator peptide, *ARCIMBOLDO*, *ARCIMBOLDO_LITE*, superhelix, left-handed twist, fragment-based molecular replacement

## Abstract

Nuclear hormone receptors are cytoplasm-based transcription factors that bind a ligand, translate to the nucleus and initiate gene transcription in complex with a co-activator such as TIF2 (transcriptional intermediary factor 2). For structural studies the co-activator is usually mimicked by a peptide of *circa* 13 residues, which for the largest part forms an α-helix when bound to the receptor. The aim was to co-crystallize the glucocorticoid receptor in complex with a ligand and the TIF2 co-activator peptide. The 1.82 Å resolution diffraction data obtained from the crystal could not be phased by molecular replacement using the known receptor structures. HPLC analysis of the crystals revealed the absence of the receptor and indicated that only the co-activator peptide was present. The self-rotation function displayed 13-fold rotational symmetry, which initiated an exhaustive but unsuccessful molecular-replacement approach using motifs of 13-fold symmetry such as α- and β-barrels in various geometries. The structure was ultimately determined by using a single α-helix and the software *ARCIMBOLDO*, which assembles fragments placed by *PHASER* before using them as seeds for density modification model building in *SHELXE*. Systematic variation of the helix length revealed upper and lower size limits for successful structure determination. A beautiful but unanticipated structure was obtained that forms superhelices with left-handed twist throughout the crystal, stabilized by ligand interactions. Together with the increasing diversity of structural elements in the Protein Data Bank the results from TIF2 confirm the potential of fragment-based molecular replacement to significantly accelerate the phasing step for native diffraction data at around 2 Å resolution.

## Abstract 2

A peptide fortuitously crystallized to form a motif exhibiting 13-fold non-crystallographic symmetry as judged by self-rotation function analysis. Molecular-replacement phasing used a small α-helix as the search model and was only successful with the recently deployed *ARCIMBOLDO_LITE*.

## 1. Introduction   

**Figure 1:**
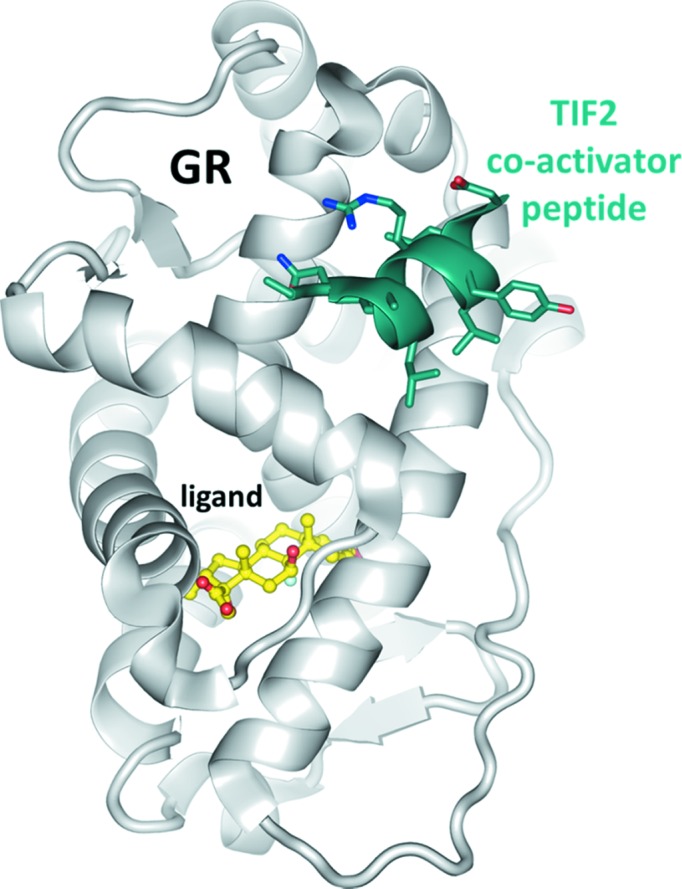
Structure of GR. The receptor (PDB entry 3mnp, Seitz *et al.*, 2010 ▶; grey ribbon) is an all-helical protein with a central solvent-excluded ligand-binding cavity and a lateral surface binding site for the co-activator. The steroid ligand and co-activator peptide TIF2 are coloured yellow and dark green, respectively. There are no direct contacts between the two molecules. The sequence of the co-activator peptide visible in this structure is NALLRYLLD.

Nuclear hormone receptors constitute a superfamily of ligand-activated transcription factors that includes the mineralo­corticoid receptor, oestrogen receptor, progesterone receptor, androgen receptor, vitamin D receptor, thyroid hormone receptor, peroxisome proliferator-activated receptors and glucocorticoid receptor (GR). Together with progesterone receptor, androgen receptor and mineralocorticoid receptor, GR belongs to the oxosteroid hormone receptor family. Upon binding of a steroid ligand such as cortisol, prednisolone or dexamethasone, GR detaches from its cytosolic complex with the chaperone HSP90, dimerizes and translocates into the nucleus where it interacts with a co-regulator. Depending on whether transcription is activated or repressed, the co-regulator can be TIF2 (transcriptional intermediary factor 2) or NCoR (nuclear receptor co-repressor). Genes regulated by GR are involved in sugar metabolism, cell differentiation, inflammation and immunosuppression (Newton, 2000 ▶). For example, the GR ligand dexamethasone has been used for decades as an anti-inflammatory and immunosuppressant and is listed by the World Health Organization as an essential medicine (http://www.who.int/medicines/publications/essentialmedicines). GR is also a potential drug target for diabetes (Jacobson *et al.*, 2005 ▶), rheumatoid arthritis (Laan *et al.*, 1999 ▶), allergic diseases (Barnes, 1999 ▶) and leukaemia (Renner *et al.*, 2003 ▶). For structure-based drug design, it is sufficient to consider the ligand-binding domain (Wurtz *et al.*, 1996 ▶) of GR in complex with a small part of the co-regulator, usually a peptide of nine to 15 residues that for the most part forms an α-helix when bound to GR (Fig. 1 ▶).

During the GR drug-design program a crystal was obtained that could not be phased by any of the known structures of GR (Bledsoe *et al.*, 2002 ▶; Kauppi *et al.*, 2003 ▶; Biggadike *et al.*, 2008 ▶, 2009 ▶; Madauss *et al.*, 2008 ▶; Suino-Powell *et al.*, 2008 ▶; Schoch *et al.*, 2010 ▶; Seitz *et al.*, 2010 ▶; Carson *et al.*, 2014 ▶; He *et al.*, 2014 ▶; Edman *et al.*, 2014 ▶), indicating that GR was not part of the crystal. Analysis of the crystal confirmed the absence of GR and the presence of the co-activator peptide TIF2 only. The diffraction data revealed intriguing 13-fold rotational symmetry, leading to the generation of a number of trial models for molecular replacement, all of which ultimately turned out to be wrong. Using an ideal α-helix as the search model, the recently deployed *ARCIMBOLDO* software (Sammito *et al.*, 2014 ▶; Millán *et al.*, 2015 ▶) in its ‘lite’ version – which is easy to install on a single workstation and which does not need setup of a scheduling grid – was able to successfully pre-assemble partial solutions from *PHASER* (McCoy *et al.*, 2007 ▶) such that they could be used for density modification and chain tracing in *SHELXE* (Sheldrick, 2010 ▶). The minimal model requirements for structure determination are reported, including the fact that a slightly too long or too short helix will result in failure. Marked differences in helix length are apparent when comparing the structures of the co-activator peptide bound to GR and in the tridecameric assembly. The TIF2 problem is captivating crystallographically and the success in structure determination using minimal prior information instils hope that other enigmatic data sets corresponding to unanticipated structures can be phased using similar approaches.

## 2. Materials and methods   

### 2.1. Peptide and protein preparation   

Human GR was prepared as described previously (Schoch *et al.*, 2010 ▶). The final concentration prior to crystallization was 16 mg ml^−1^ in 50 m*M* Tris–HCl pH 7.5, 10% glycerol, 0.3 *M* NaCl, 2 m*M* TCEP, 0.5% of the cholic acid derivative CHAPS (equivalent to 80 m*M*). The co-activator peptide is derived from TIF2 and was synthesized as acetyl-KENALLRYLLDKD-CONH_2_, *i.e.* with the N-terminus acetylated and the C-terminus amidated (molecular weight 1632 g mol^−1^). The electron density later confirmed that this sequence is wrong and the first two residues KE of the peptide are in fact EK.

### 2.2. Crystallization, crystal analysis and data collection   

20 µl of 11 m*M* TIF2 in 50 m*M* Tris–HCl pH 7.5 was mixed with 200 µl of 16 mg ml^−1^ GR and set up for sitting-drop vapour-diffusion crystallization at 295 K. Complex and reservoir were mixed in a 1:1 ratio with 1–2 µl drop volume. Crystals were obtained from 2 *M* (NH_4_)_2_SO_4_, 5–10% glycerol, 0.1 *M* citric acid pH 3.5 and were analysed by HPLC in H_2_O + 0.1% TFA (trifluoroacetic acid) on a Poroshell 300SB-C8 (Agilent) using a 10–70% linear gradient to acetonitrile + 0.08% TFA over 4 min at 1 ml min^−1^ flow rate. The signal was monitored at 214.4 nm, *i.e.* at the backbone amide absorption. Crystals were cryo-protected with 16% glycerol, 1.6 *M* (NH_4_)_2_SO_4_, 80 m*M* citric acid pH 3.5. Diffraction data were collected at 100 K on beamline PX-II at the Swiss Light Source using a MAR CCD 225 detector, and integrated and scaled in space group *C*2 with *DENZO* (Otwinowski & Minor, 1997 ▶) and *SADABS* (Bruker), respectively (Table 1 ▶). Assuming one GR/TIF2 complex in the asymmetric unit the Matthews coefficient (Matthews, 1968 ▶) is 2.5 Å^3^ Da^−1^. After structure determination the true Matthews coefficient turned out to be 2.8 Å^3^ Da^−1^ (33 kDa molecular weight, including H atoms, ligands, water and sulfates) with a solvent content of 55.6%. No anomalous information that could have helped in phasing was detected in the data as judged by analysis with *XPREP* (Bruker). Patterson maps and rotation functions were calculated with *FFT* and *POLARRFN*, respectively (Winn *et al.*, 2011 ▶). Model building was done with *COOT* (Emsley *et al.*, 2010 ▶) and figures were drawn with *PyMOL* (Schrödinger).

## 3. Results   

**Figure 2:**
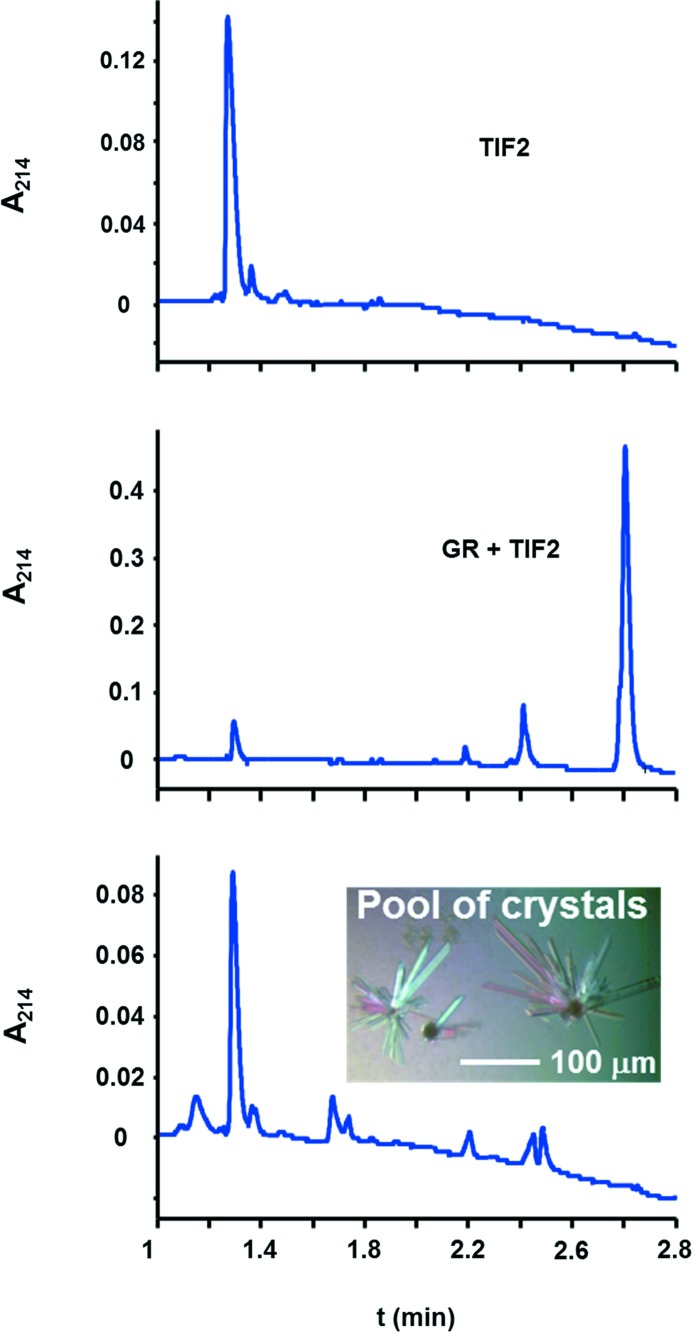
HPLC analysis of the supposed GR/TIF2 complex crystals reveals the presence of TIF2 peptide only. The top panel establishes that TIF2 elutes at 1.3 min. The middle panel is a mixture of TIF2 peptide and GR as used for crystallization. The largest peak at 2.7 min is GR. The peak at 2.4 min is an unknown compound, probably a contaminant as it also appeared in the bottom panel and in blank runs (not shown). The bottom panel represents the washed crystals. As no GR is present, the crystals only contain TIF2 peptide. Other signals may belong to crystallization buffer components that have not been washed away.

None of our previously determined mouse (Seitz *et al.*, 2010 ▶) and human GR structures (Schoch *et al.*, 2010 ▶) could be used to phase the diffraction data. Since nuclear receptors are known to exhibit large conformational changes (Veleiro *et al.*, 2010 ▶), a selenomethionine-modified GR was prepared in order to obtain experimental phases. However, the crystals did not exhibit the expected fluorescence excitation for selenium (data not shown), which raised the first suspicion that the receptor might not even be part of the crystals. Native crystals were washed and subjected to mass spectrometry, which confirmed the presence of TIF2, but no GR was detected (data not shown). To exclude the possibility of inefficient ionization of GR in the mass spectrometer, HPLC as an independent method was performed on washed crystals (Fig. 2 ▶) and compared to the elution properties of the putative constituents GR and TIF2. The HPLC analysis confirmed the absence of GR and only TIF2 was detected in the crystals. These observations are also in accord with the absence of UV-induced fluorescence in the crystals as only GR but not TIF2 contains tryptophan. The volume of the *C*2 asymmetric unit is large enough to host up to 22 peptide molecules of 1.6 kDa at a Matthews coefficient of 2.5 Å^3^ Da^−1^. A large number of molecules would probably render structure determination somewhat difficult, even if the structure of the motif were known. Indeed, initial attempts to use the TIF2 structure from previously determined GR complexes, a short α-helix of nine to ten residues (Fig. 1 ▶), as a search model in *PHASER* did not yield a solution that was interpretable by eye or that could be extended into an interpretable map by *SHELXE*.

### 3.1. Search models in accord with the self-rotation function   

**Figure 3:**
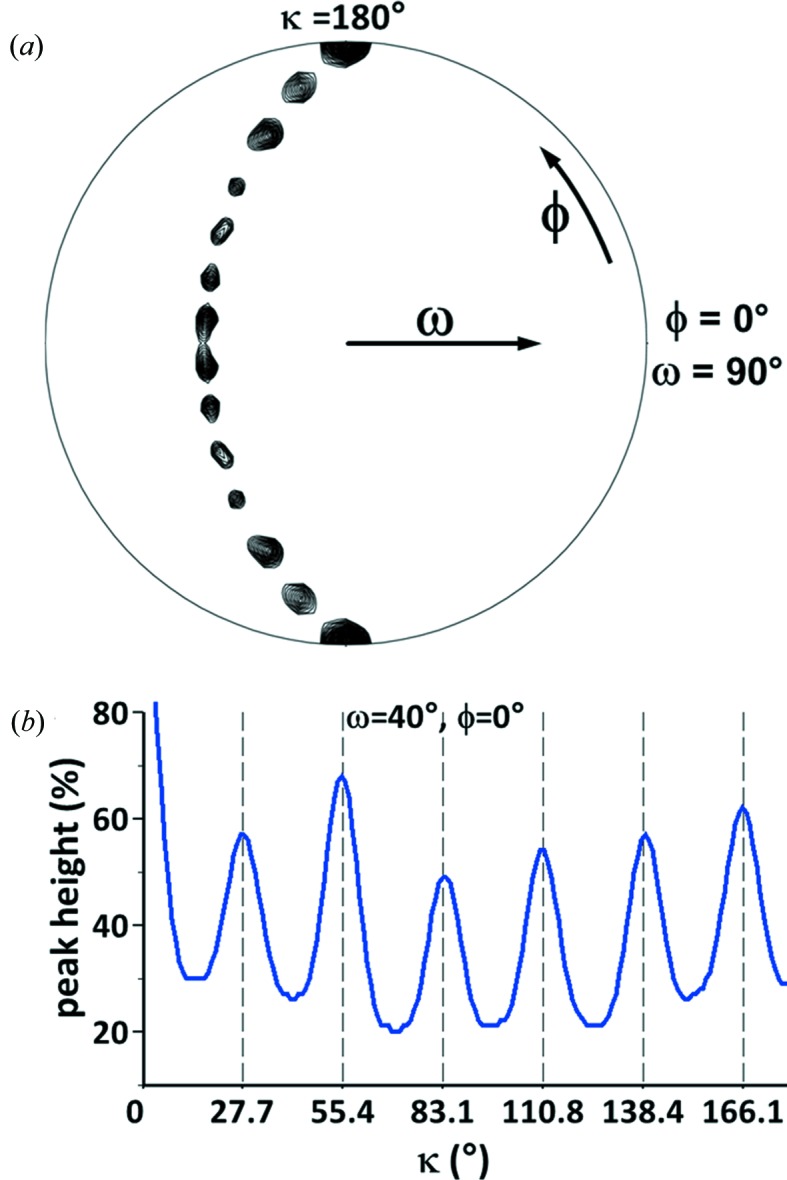
Self-rotation function analysis of the diffraction data. (*a*) The stereographic projection of the κ = 180° section of the self-rotation function calculated at 1.82 Å resolution in space group *C*2 was plotted at a contour level of 40% of the origin peak. The crystallographic twofold axis is at ω = 90°, ϕ = 90°. Six additional twofold axes are visible which form a crescent. (*b*) Signal of a recurrent peak at ω = 40°, ϕ = 0° as a function of κ that is not visible in (*a*). The peaks follow the term (*n* × 360°)/13 with the integer *n* ≤ 6, indicating 13-fold NCS. Combination of this axis with the crystallographic twofold axis leads to the twofold NCS axes visible in (*a*).

If the crystals contain several copies of a small motif, some type of non-crystallographic symmetry (NCS) would have been expected. Apart from the crystallographic peak arising from *C*-centring, the Patterson function (Patterson, 1935 ▶) was featureless, which excludes translational NCS. By contrast, a self-rotation function analysis revealed the presence of a 13-fold rotational NCS (see Fig. 3 ▶ and the movie in the supporting information), which is consistent with 13 entities arranged in a ring-like structure. The corresponding Matthews coefficient would be rather large, 4.3 Å^3^ Da^−1^, if the crystal were composed of the 1.6 kDa TIF2 peptide only. Because the diffraction data extend to a resolution of 1.82 Å (Table 1 ▶), the crystal should be packed rather tightly, indicating that either another component is part of the 13-fold entity or there is scattering mass that does not exhibit 13-fold symmetry.

**Figure 4:**
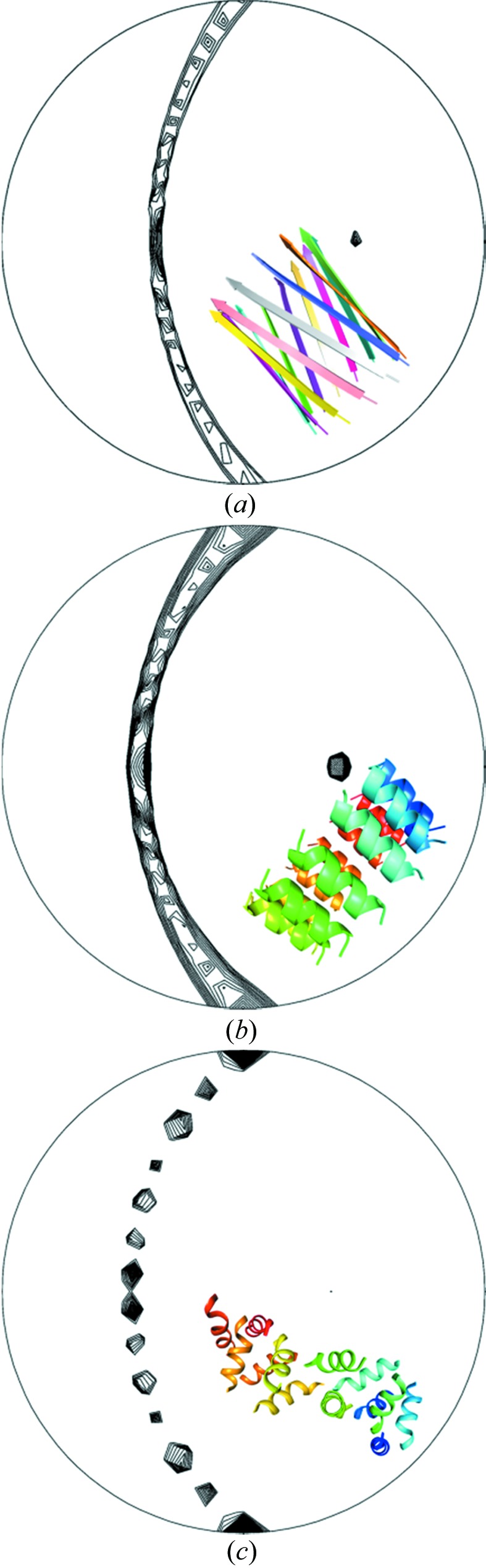
Stereographic projections of the κ = 180° section of the self-rotation functions from (*a*) a 13-mer parallel β-barrel, (*b*) a tridecameric α-barrel from PDB entry 2x2v and (*c*) the refined superhelical TIF2 structure. The molecules in (*a*) and (*b*) were aligned with the NCS axis at ω = 40°, ϕ = 0° to facilitate comparison with the diffraction data (Fig. 3*a*) and the final model in (*c*). The regular barrels have more internal symmetry elements than the true structure. Of note is the presence of the 13-fold NCS axis in this κ section, but its absence in the correct structure in (*c*). Calculations were done as in Fig. 3(*a*) except that for the representations (*a*) and (*b*) the initial contour levels were raised to 90% and 70% of the *C*2 crystallographic peak in order to better visualize the twofold axes.

The 13-fold NCS inspired the construction of search models for molecular replacement of the same symmetry. The ring-like shape of the 13-mer indicated by the self-rotation function could possibly be a planar barrel. In case there was a translational component (for which the self-rotation is insensitive) this translation must not result in a pseudo-translational NCS because the Patterson function does not support it (see above). Such a barrel would have to have its constituents parallel to each other since only even-numbered barrels can have an entirely anti-parallel arrangement. The termini in a parallel barrel are aligned, an unlikely situation for a charged peptide. However, as the TIF2 peptide is N-terminally acetyl­ated and C-terminally amidated, no Coulomb repulsion is present and a parallel arrangement is possible. Short peptides in solution cannot be assumed to fold into the structures they adopt when bound to a larger protein, so any regular arrangement of the peptide with a 13-fold symmetry is a possibility. Peptides have been observed to form β-type fibril structures (Smith, 2012 ▶; Tycko & Wickner, 2013 ▶) and there is a multitude of parallel β-barrel substructures with different radii and tilt angles in the PDB (Protein Data Bank) that have up to 39 strands in the major vault protein (Casañas *et al.*, 2013 ▶). A model for a tridecameric parallel β-barrel was constructed and, when aligned with the NCS axis, it roughly reproduces the self-rotation function of the diffraction data (Fig. 4 ▶ *a*). Although the similarity between the self-rotation function sections is less than hoped for, we constructed 6480 barrels with varying radii (11–19 Å), strand rotations (0–355°, even if this meant breaking hydrogen bonds) and tilt angles (0–45°). All models failed to result in a molecular-replacement solution based on negative log-likelihood gains in *PHASER*, which essentially means that they performed worse than a random assortment of atoms.

Analysis of the Wilson plot (Morris *et al.*, 2004 ▶) for secondary-structure content (Popov *et al.*, personal communication) suggested a high α-helical content of the structure (55 ± 15%), while the content of β-strands was predicted to be low (*circa* 10 ± 10%). Thus, α-helical barrel structures were tested next as possible models for molecular replacement. Such barrels produce similar self-rotations (Fig. 4 ▶ *b*) albeit with more symmetry elements present compared to the diffraction data (Fig. 3 ▶ *a*). The Crick parameters for α-helical barrels (Crick, 1953 ▶) were used to generate a few hundred parallel tridecameric barrels of various radii and helix tilt angles [program provided by J. Holton using the formulation from Harbury *et al.* (1995 ▶)] for molecular-replacement searches. This approach was successful previously in the structure determination of the four-stranded coiled-coil A-domain of the voltage-gated potassium channel Kv7.4 (Howard *et al.*, 2007 ▶). α-Helical barrels are usually not as wide as β-barrels, unless they are part of a larger assembly. For example, the ATP synthase rotor ring (PDB entry 2x2v, Preiss *et al.*, 2010 ▶) has 13-fold symmetry with two nested barrels of different diameter. After none of the barrels resulted in a molecular-replacement solution, it was realized that a single α-helix also exhibits 13-fold internal symmetry: the 3.6_13_-helix has 13 atoms in a hydrogen-bonded ring as the repeating pattern. Alignment of a single α-helix with the NCS axis in the crystal setting of the data produces similar features in the self-rotation function compared to the diffraction data (not shown). However, the cell volume is too large to host just a single long α-helix, and several parallel helices should have manifested as a repeating pattern in the self-Patterson map, similar to what is observed with DNA (Egli *et al.*, 2007 ▶).

At this point, although the scattering mass of the search model was <10% of the asymmetric unit, *PHASER* molecular-replacement searches for 13 copies of a single α-helix in any orientation were started with ideal α-helices of varying lengths. Although a solution with a high LLG (log-likelihood gain) value of 443 was obtained for a search with a decamer helix and the model did not exhibit excessive clashes, the electron-density maps were uninterpretable and did not improve after density modification with *SHELXE*. Expert handling of the programs’ parameters might have resulted in a more favourable outcome, but in our hands, the *ARCIMBOLDO* approach described in the following was straightforward.

### 3.2. Structure determination with *ARCIMBOLDO* and model requirements   

*ARCIMBOLDO* (Rodríguez *et al.*, 2009 ▶; Sammito *et al.*, 2014 ▶) is a program that combines the programs *PHASER* and *SHELXE*. Originally intended for *ab initio* phasing at atomic resolution (1.2 Å or better), accurate secondary-structure fragments such as ideal polyalanine α-helices can be placed using *PHASER* and are then selected and pre-assembled as starting coordinates for several cycles of automated chain tracing after density modification in *SHELXE*.

**Figure 5:**
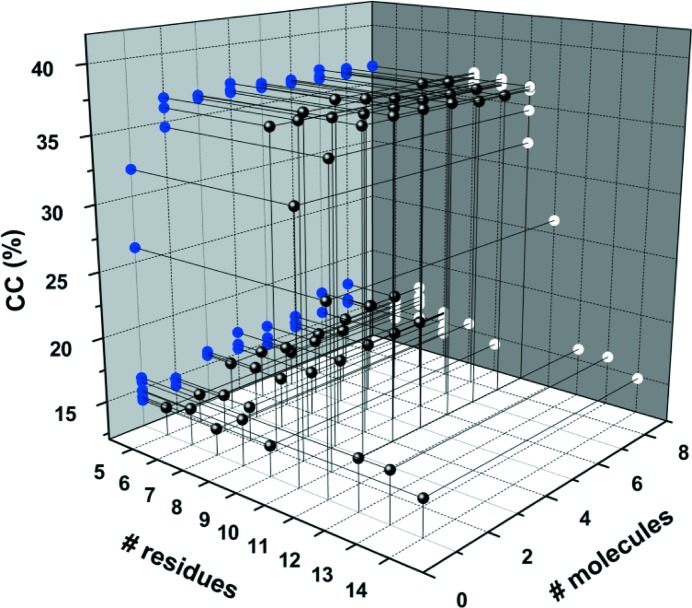
Length requirements for the ideal polyalanine α-helix search model. Helices between five and 14 residues were used as models, and one to eight copies were searched for in *PHASER*, followed by three cycles of density modification and automated polyalanine chain tracing in *SHELXE*. All calculations were done at the maximum resolution of 1.82 Å. The solvent content was set to 0.65, slightly higher than expected in order to help solvent flattening, and in the auto-tracing α-helices were searched for in all cycles (-q option). The NCS option in *SHELXE* is available for substructures but not for tracing and hence was not applicable here. Helices between eight and 11 residues are suitable search models as judged by CC > 25%. The most extensive search performed was for nine fragments of a decamer helix, which yielded a final LLG of 576. For improved legibility the data points (black spheres) are projected onto the grey walls of the plot (blue and white dots).

A structure is possibly determined if the *SHELXE* map correlation coefficient (CC) exceeds 25%, although the threshold depends on the resolution and presence of translational NCS. The ‘lite’ version of *ARCIMBOLDO* was used on a computer with 16 Intel Xeon CPUs (E5-2670, eight cores @ 2.60 GHz) and with 132 GB RAM running CENTOS 6.5 to yield a traced (98 residues) solution within an hour using an ideal polyalanine α-helix of ten residues and all *B* values set to 30 Å^2^ as the initial model (Fig. 4 ▶ *c*). The model requirements for success were then systematically tested by varying the helix length and the number of molecules that were searched for (Fig. 5 ▶). Interestingly, there are sharp boundaries between success and failure. The smallest ideal helix that can yield a solution (five residues was the lower limit tested) is eight residues when at least two molecules are searched for in *PHASER*. The resulting CC is 36%. The same conditions apply to a nonamer helix, but for helices of seven or less residues no solution was obtained, even if eight molecules were searched for in *PHASER* (12 h CPU time). On the other hand, helices with ten or 11 residues require only a single copy to be found in order to enable chain tracing in *SHELXE*, which corresponds to 3.5% and 3.8% of the total peptide scattering mass (1443 atoms). Helices of 12 and more residues fail to produce a solution, both with and without using packing considerations as a rejection criterion for model placement. Taken together there is both a lower and an upper limit for a suitable search model. Because a single residue can make the difference between success and failure, varying the size of the molecular fragment that is used in *ARCIMBOLDO* may prove useful for other cases, computing power permitting. Interestingly, a single helix from the refined structure is a valid search model without invoking *ARCIMBOLDO*, but the TIF2 peptide from other GR/TIF2 complexes truncated to eight, nine or ten residues is not. The main-chain atom r.m.s.d. (root mean square distance) between these TIF2 conformations is 0.7 Å (see below for conformational differences), placing a lower limit for model precision in this case. The main-chain atom r.m.s.d.s of the 13 refined TIF2 chains differ from an ideal helix by only 0.38 ± 0.03Å, indicating that somewhere between 0.4 and 0.7 Å r.m.s.d. between the coordinates of the search model and the true structure a molecular replacement will be successful.

The strategy underlying *ARCIMBOLDO* is to locate a partial, yet very accurate, substructure and expand it through iterative density modification and autotracing. Previous experiences with *SHELXE* expansion (Thorn & Sheldrick, 2013 ▶) have shown that a large penalty is paid for incorrect parts in this substructure. Much better results are obtained with a smaller, error-free model than for a more complete but inaccurate model. In other words, the search for a good partial substructure followed by expansion of such solutions with *SHELXE* may be superior to an attempt to generate a complete model by placing all 13 helices first and then running density modification and autotracing. In addition, correct partial solutions may be discarded by the packing test if the next copy is incorrectly placed. After placement of each new fragment, an initial CC (Fujinaga & Read, 1987 ▶) is calculated and optimized by sequentially omitting every amino acid in the partial model and eliminating them if this leads to CC improvement (Sheldrick & Gould, 1995 ▶). Models are scored after this so-called omit CC and a number of partial structures equal to the number of physical cores in the computer minus one are sent to expansion. For the TIF2 case, even in a search for six helices the best solution was already reached with a substructure obtained after placement of only four fragments (see Fig. S2 in the supporting information).

### 3.3. TIF2 forms a superhelix with a left-handed twist   

**Figure 6:**
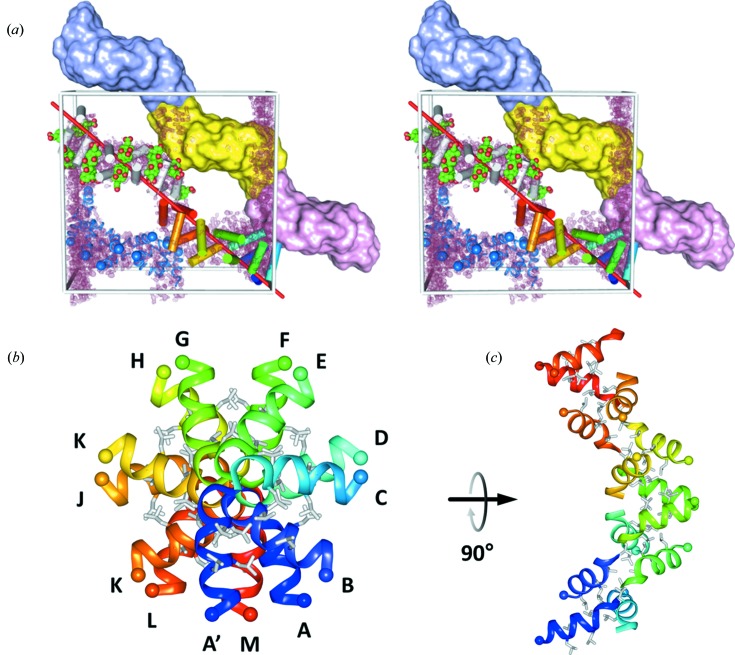
Superhelical TIF2 structure and packing in the *C*2 unit cell. (*a*) The *C*2 unit cell is shown in cross-eyed stereo as a grey box with the origin at the bottom right and its four asymmetric units. One asymmetric unit consists of 13 short helices of the TIF2 peptide (coloured cylinders) that are arranged around the NCS axis, which is shown as a red line. Another asymmetric unit centred on the NCS axis is shown in grey with space-filling models (green) of the cholic acid molecules that wedge between the helices. The asymmetric units combine to form a continuous left-handed superhelix that traverses the crystal, which is well visible through a surface representation of three individually coloured asymmetric units. The fourth asymmetric unit is coloured blue and the N-termini of the helices are marked by a sphere, showing that the arrangement of TIF2 helices is not all parallel as assumed in the models in Figs. 4 ▶(*a*) and 4 ▶(*b*). The *SHELXE*-derived electron density is contoured at 1 r.m.s.d. for the whole unit cell. (*b*) View of the asymmetric unit projected along the NCS axis with the individual chains labelled. The 14th helix A′ shown in dark blue serves to highlight the repeating pattern in the superhelix. The N-termini are marked by spheres and point to the outside of the superhelix. Leucine side chains that construct the hydrophobic core are drawn as grey stick models. (*c*) View 90° rotated relative to (*b*).

**Figure 7:**
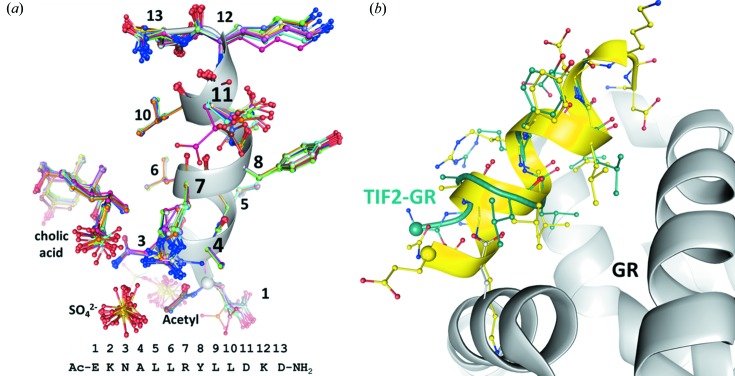
TIF2 conformations in the superhelix and when bound to GR. (*a*) Superposition of all 13 protomers. The residues are numbered according to the sequence given at the bottom. The main-chain torsion angles of residues 12 and 13 deviate from α-helical geometry. Because of weak electron density, these residues were modelled with half occupancy. Clashes of these residues with the same residues of neighbouring helices indicate that the crystal actually contained a mixture of peptides. (*b*) The GR/dexamethasone/TIF2 complex (Seitz *et al.*, 2010 ▶) is superimposed with one representative protomer (coloured yellow) from the superhelix. GR is coloured grey and the TIF2 peptide in complex with GR is shown in dark green. The N-termini of the peptides are marked by spheres. The N-terminal part of TIF2 cannot adopt an α-helical conformation when binding to GR due to clashes with the receptor.

Placement of the 13 TIF2 helices into closest proximity revealed their arrangement as a superhelix of outer diameter 42 × 46 Å. The superhelix has a left-handed twist and is extended by crystallographic symmetry, hence traversing the crystal (Fig. 6 ▶ *a*). The height of a single turn is 66 Å with 13 repeats per turn. The projection of the asymmetric unit along the NCS axis is a barrel (Fig. 6 ▶ *b*), albeit very different compared to the parallel α-helical barrels considered above. The helices are not arranged parallel to each other but are organized in pairs which point their C-termini towards each other and the N-termini are located on the surface of the superhelix (Fig. 6 ▶). This non-parallel arrangement of protomers may contribute to the absence of self-Patterson peaks and would have been difficult to anticipate from NCS analyses. The repeating unit in the superhelix is thus a pair of α-helices, one of which is shared by two individual turns. There are two pairs of leucine residues in the TIF2 sequence, Leu5/6 and Leu9/10, which interlace to form a continuous hydrophobic core in the superhelix (grey sticks in Figs. 6 ▶ *b* and 6 ▶ *c*). A similarly extended hydrophobic core is present in the packing of the P22 tailspike adhesin β-helix (Simkovsky & King, 2006 ▶), in armadillo repeats (Reichen *et al.*, 2014 ▶) and in amyloid-β structures (Colletier *et al.*, 2011 ▶). Together with a cholic acid ligand interaction (see below) this interdigitation of aliphatic residues seems to be the driving force for generating the TIF2 superhelix. The individual helices are quite similar to each other and superimpose with r.m.s.d. values of 0.12 ± 0.03 Å and 0.22 ± 0.06 Å, respectively, for secondary-structure matching of the main-chain atoms and for least-squares matching of all atoms including side chains (Fig. 7 ▶ *a*). A central core of eight residues (NALLRYLL) is almost invariant among the protomers. The largest conformational differences are seen in the two C-terminal residues, which deviate from ideal α-helical geometry, the tips of the Glu1 and Arg7 side chains, and the carboxylate group of cholic acid (see below). Two sulfate ions are present per protomer; one is located close to the N-terminus and neutralizes the helix dipole, and the other is bound to the guanidinium side chain of Arg7. The acetyl group at the N-terminus of the TIF2 peptide continues the helical hydrogen-bonding pattern, effectively serving as a helix cap (Fig. 7 ▶ *a*).

### 3.4. TIF2 has different conformations when bound to GR or when assembled into a superhelix   

When bound to GR the TIF2 sequence NALLRYLL forms an eight-residue α-helix with frayed ends. In contrast, when crystallized alone the TIF2 peptide forms an α-helix over its entire length less the C-terminal aspartyl amide, but including the N-terminal acetyl group. The two TIF2 conformations superimpose with a main-chain atom r.m.s.d. of 0.7 Å and have their N-termini 4.5 Å away from each other (spheres in Fig. 7 ▶ *b*). The conformation of TIF2 that is present in the superhelix would clash with residues from GR, explaining why the helix of the co-activator peptide must be shorter when bound to the receptor. There is currently no crystal structure available for the entire co-activator (the entire TIF2 has 1464 residues) or a structural domain encompassing the TIF2 motif that recognizes GR. Thus, at present it cannot be determined whether the co-activator helix has to partially melt in order to bind to GR or whether its conformation in the context of the co-activator is already the same as that seen in the GR/TIF2 complex.

### 3.5. The TIF2 superhelix is stabilized by a steroid ligand   

**Figure 8:**
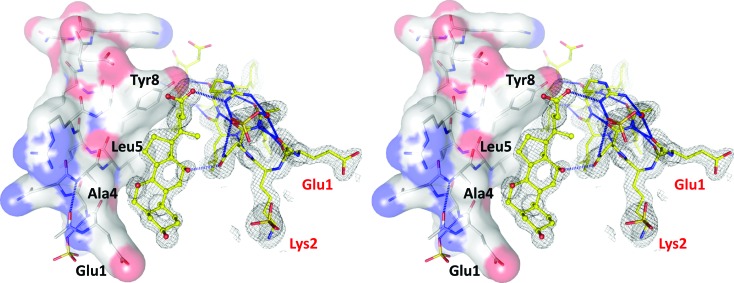
Sandwiching of cholic acid between protomers stabilizes the superhelix. The cross-eyed stereo image shows density-modified but unbiased (*i.e.* before building of the cholic acid) electron density at the 2 r.m.s.d. level as a grey mesh. The density for the two N-terminal residues Glu1 and Lys2 reveals the error in the sequence of the synthetic peptide (red labels). The hydrophobic concave surface into which cholic acid binds is lined by Glu1, Ala4, Leu5 and Tyr8.

The high quality of the *SHELXE* electron density allowed detection of an error in the TIF2 peptide sequence used for crystallization (Fig. 8 ▶). The first two residues are swapped, possibly due to a typo that occurred when the peptide synthesizer was programmed. In addition, non-proteinaceous electron density identified a molecule that is wedged between two adjacent TIF2 helices and that mediates contact between them by means of hydrogen bonds and van der Waals interactions (Fig. 8 ▶). From the *SHELXE* density alone it was clear that the ligand is a steroid, which was unanticipated but is easily explained by the buffer component CHAPS, an amide of cholic acid. No electron density for the amino part of this detergent was detected, indicating either disorder or a hydrolytic event that generated cholic acid. Quantitative mass-spectrometric analysis of CHAPS revealed the presence of 40 p.p.m. cholic acid, corresponding to 49 µ*M* in the crystallization buffer. Thus, in principle there is enough contaminating cholic acid to explain its presence in the crystal structure. The cholic acid composes about one quarter of the total scattering mass.

Cholic acid is a convex molecule that fits into a concave surface patch lined by the methylene groups of Glu1, the side chains of Ala4 and Leu5, and the aromatic face of the Tyr8 side chain. Connections to the second helix of the sandwich are made by two hydrogen bonds. A hydroxyl group of the steroid binds to the carbonyl group of the Asn3 carboxamide, and the carboxylate of cholic acid engages in a (possibly charged) hydrogen bond with the guanidinium group of Arg7. The aliphatic parts of the Leu6, Arg7 and Leu10 side chains also form a few van der Waals interactions with cholic acid. The interactions between cholic acid and TIF2 are serendipitous and unlikely to bear biological significance for nuclear receptor biology. When bound to GR, the ligand and co-activator peptide do not directly interact (Fig. 1 ▶).

## 4. Discussion   

Superhelices are frequently observed in proteins with sequence repeats. The repeats fold into a small structural element that can be a small superhelix of either handedness. In case these structural elements are parallel a ring-shaped solenoid results, but if successive elements are incrementally rotated with respect to each other along the superhelical axis, the solenoid twists and becomes a superhelix (Kobe & Kajava, 2000 ▶). Thus, there are two superhelical parameters to be considered, the superhelicity imposed by the direction of the polypeptide chain, and the overall twist of the superhelix. Although these parameters do not seem to depend on each other, most superhelices are right-handed, both in chain direction and twist. Examples include the α-helical armadillo repeats (Conti *et al.*, 1998 ▶; Huber & Weis, 2001 ▶) and tetratricopeptide repeats (Jínek *et al.*, 2004 ▶; Yuzawa *et al.*, 2011 ▶). The α/β-structured leucine-rich repeats can also twist into a right-handed superhelix, as exemplified in the receptor kinase BRI1 (Hothorn *et al.*, 2011 ▶). In contrast, the α-helical HEAT repeats (Cingolani *et al.*, 1999 ▶) can induce left- and right-handed superhelical twists. Currently, the only example of a left-handed α-helical solenoid forming a right-handed superhelix is the Zurdo domain of human mitochondrial regulator mTERF (Jiménez-Menéndez *et al.*, 2010 ▶). Pronounced left-handed twists in superhelices are rare and limited to examples such as the α/β ankyrin repeats in p53 binding protein (Gorina & Pavletich, 1996 ▶) and the parallel β-helix in pectate lyase (Lietzke *et al.*, 1996 ▶). The strong left-handed twist in TIF2 therefore comes as an exception, being the only all α-helical motif that assembles into such a superhelix. The TIF2 peptide protomers associate in pairs with their N-termini pointing away from the superhelical axis. Therefore, no pseudo-solenoid path can be constructed as this requires alternating N- and C-termini in close proximity. More structures of α-helical peptides are required to denote the rules governing their superhelical assemblies.

Peptide assemblies are prominent in a number of neurodegenerative disorders such as Alzheimer’s disease, Parkinson’s disease, spongiform encephalopathies and Huntington’s disease (Gadad *et al.*, 2011 ▶). The aggregates are formed by peptides that are derived from normal cellular proteins. The peptides do not share sequence homology but exhibit significant flexibility in solution and fold into β-type structures, especially under conditions of low pH, high salt concentration and high peptide concentration (Murphy, 2002 ▶). Superhelical structures can also be adopted by these peptides. For instance, the β-amyloid peptide 42 forms a superhelix where helical β-sheets wrap around each other (Stroud *et al.*, 2012 ▶). The superhelical structure formed by the TIF2 peptide raises the possibility that the α-helical conformation of peptides can promote aggregates *in vivo* as well. While the β-amyloid peptide in Alzheimer’s disease encompasses 42 residues, the smallest toxic peptide with only 14 residues is the prion protein PrP 113–126 (Brown, 2000 ▶; Murphy, 2002 ▶), which is on the same scale as the TIF2 peptide. In addition, an N-terminal extension of the PrP 109–122 by five residues changes the preferred structure from β-sheet to α-helical (Nguyen *et al.*, 1995 ▶). Thus, small peptides are in principle able to associate into large stable aggregates of either secondary-structure type. The TIF2 structure is stabilized by cholic acid, a bile acid precursor that is also found in cerebrospinal fluid (Ogundare *et al.*, 2010 ▶). Without speculating too much, it is conceivable that endogenous ligands could stabilize the quaternary structure of α-helical peptide aggregates. In turn, such a stabilizing small-molecule drug could help inhibit the fibrillation cascade that leads to β-type deposits.

## 5. Conclusions   

For the last two decades, anomalous diffraction methods such as MAD (multi-wavelength anomalous diffraction), SAD (single-wavelength anomalous diffraction) and SIRAS (single isomorphous replacement with anomalous signal) have contributed the majority of *de novo* phased structures. In the absence of anomalous signal, however, direct methods and molecular replacement remain the only avenues to obtain phase information, and for most biological samples, direct methods are out of the question because the crystals do not diffract to atomic resolution. The data used in this study were collected in 2006 and could not be phased despite considerable effort including preparation of selenomethionine-modified GR (which is absent) and somewhat extensive (and futile) molecular-replacement searches using models derived from self-rotation function analyses. During the past eight years, neither crystals nor starting materials for this project have been available, so molecular replacement remains the only viable approach. Software development and Moore’s law allowed the computationally expensive combination of molecular replacement with chain tracing, which led to facile structure determination shortly after the release of the simplified version *ARCIMBOLDO_LITE* (Millán *et al.*, 2015 ▶). We suspect that there are many resilient data sets like the one discussed here in crystallography laboratories, which might be phased by molecular replacement. The extension of the *ARCIMBOLDO* method from secondary-structure elements to tertiary structures, either probing fragments from distant homologues with *ARCIMBOLDO_SHREDDER* (Sammito *et al.*, 2014 ▶), or using canonic fragments as model structures with *ARCIMBOLDO_BORGES* (Sammito *et al.*, 2013 ▶), together with the probably valid assumption that most macromolecular structural elements are nowadays represented in the PDB, could significantly speed up the phasing step provided that native diffraction data to about 2 Å resolution are available. To the best of our knowledge, there are currently 30 previously unknown structures that have been determined by *ARCIMBOLDO* methods. The release of the single-machine implementation *ARCIMBOLDO_LITE* may contribute to popularizing this phasing avenue (Rodríguez *et al.*, 2012 ▶). It could also possibly lead to a shift in the preferred method to phase unknown structures away from anomalous data to molecular replacement of native data using small structural elements, including DNA libraries (Pröpper *et al.*, 2014 ▶), as seeds.

## Supplementary Material

PDB reference: 4wg0

Supporting Movie. DOI: 10.1107/S2052252515000238/mf5008sup1.avi

Supplementary Fig. S2. DOI: 10.1107/S2052252515000238/mf5008sup2.html

## Figures and Tables

**Table 1:** Data collection and refinement statistics for 4wg0 Unless noted otherwise, values in parentheses correspond to the highest resolution shell.

Data collection	
Wavelength ()	0.9722
Resolution range ()	45.21.82 (1.891.82)
100% criterion ()†	1.85
Range/increment ()	245/1.0
Mosaicity ()	0.69 0.14
Space group	*C*2
Cell dimensions (,)	*a* = 95.9, *b* = 37.8, *c* = 101.4, = 96.8
Total reflections	131943 (11128)
Unique reflections	32046 (3053)
Multiplicity	4.1 (3.6)
Completeness (%)	97.5 (93.1)
*R*_merge_/*R*_meas_ ‡	0.05/0.06
CC_1/2_/CC*‡	0.999 (0.852)/1 (0.959)
Average *I*/(*I*)	15.1 (3.0)
Wilson *B* (^2^)	25.4
|*E* ^2^ 1|‡ §	0.784 (0.736/0.541)
Mean *L* ^2^ ‡ §	0.352 (0.333/0.200)
Refinement	
Resolution range ()	45.21.82 (1.891.82)
No. of work reflections	32008 (2621)
No. of test reflections	1605 (136)
*R*_cryst_ (%)¶	18.5 (25.5)
*R*_free_ (%)¶	21.8 (29.0)
No. atoms: non-H, peptide, ligands††	2177, 1491, 559
No. residues: peptide, H_2_O, SO 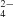 , CHD‡‡	169, 127, 24, 13
Coordinate/phase errors (/)§§	0.18/23.3
R.m.s.d. bonds/angles (/)§§	0.012/1.52
Ramachandran plot (%)¶¶	98.2/1.2/0.6
MolProbity/clashscore†††	1.91/6.0
*B* (^2^): protein, H_2_O, SO 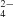 , CHD	45, 42, 82, 50

† The 100% criterion was calculated using *SFTOOLS* (Winn *et al.*, 2011 ▶) and represents the resolution in of a 100% complete hypothetical data set with the same number of reflections as the measured data.

‡ *E* values, *L* values (for acentric reflections) and *R* factors were calculated using *PHENIX* (Zwart *et al.*, 2008 ▶). *R* values and the correlation coefficients CC_1/2_ and CC* are defined in Diederichs Karplus (1997 ▶) and Karplus Diederichs (2012 ▶), respectively.

§ Values in parentheses are the expected values for untwinned and perfectly twinned data, respectively.

¶ *R*_cryst_ = 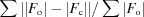, where *F*_o_ and *F*_c_ are the structure-factor amplitudes from the data and the model, respectively. *R*_free_ is *R*_cryst_ with 5% of test set structure factors.

†† Ligands are sulfate and cholic acid.

‡‡ Chains *A* and *B* have only one SO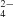 associated with them.

§§ Calculated using *PHENIX* (Zwart *et al.*, 2008 ▶).

¶¶ Calculated using *COOT* (Emsley *et al.*, 2010 ▶). Numbers reflect the percentage of amino-acid residues in the core, allowed and disallowed regions, respectively. The ill-fitting residue is Lys12 of chain *K*, which has poor electron density.

††† MolProbity score should approach the high resolution limit (Chen *et al.*, 2010 ▶). Clashscore is defined as the number of unfavourable all-atom steric overlaps 0.4 per 1000 atoms (Word *et al.*, 1999 ▶).
