# Supplementary material for: Structure of a 13-fold superhelix (almost) determined from first principles
Source: IUCrJ. 2015 Jan 27;2(Pt 2):177–87. doi: 10.1107/S2052252515000238 (PMC4392412; doi:10.1107/S2052252515000238)
Supplement: Supplementary file 2 [file m-02-00177-sup2.html]

gr\_def


# ARCIMBOLDO

Borges started at: 2014-08-10 10:03.  
  
=============================**INPUT FILE 10\_6.bor**=============================  

```
[ARCIMBOLDO]
shelxe_line = -m30 -v0 -a3 -t20 -q -s0.65
topbtf_n = 50
sampling_rotation = -1
toprnp_n = 200
pack_clashes = 0
resolution_rotation = 1.0
use_packing = True
rotation_clustering_algorithm = rot_matrices
topfrf_n = 100
force_core = -1
molecular_weight = 22000
topftf_n = 50
topexp_n = 60
toppack_1 = 10000
threshold_algorithm = 15
topfrf_1 = 1000
sigf_label = SIGF
f_label = F
fragment_to_search = 6
topbrf_n = 100
toprnp_1 = 1000
force_exp = False
topbtf_1 = 50
pack_distance = 3.0
number_of_component = 1
resolution_translation = 1.0
identity = 0.2
exclude_zscore = 0
exclude_llg = 0
rmsd = 0.2
topbrf_1 = 1000
tncs = True
resolution_refinement = 1.0
toppack_n = 10000
post_mortem = False
topexp_1 = 60
topftf_1 = 50
rncs_matl = []
name_job = gr_def
spacegroup = 
helix_length = 10
sampling_refinement = -1
sampling_translation = -1

[GENERAL]
pdb_path = 
ent_path = 
mtz_path = %(data_directory)s/sad.mtz
working_directory = %(data_directory)s/10_6
hkl_path = %(data_directory)s/sad.hkl
data_directory = gr
mtz_p1_path = 

[LOCAL]
path_local_phaser = phaser
path_local_shelxe = shelxe
```

============================================**Summary of your data**=====================================================

SPACEGROUP: C 1 2 1  
CELL DIMENSIONS: 95.87, 37.78, 101.43, 90.00, 96.84, 90.00  
RESOLUTION: 1.73  
NUMBER OF UNIQUE REFLECTIONS: 36355.00

## **2. STEP:** Locating Sequentially the Fragments

| Fragment 1 | | | | | | | | | | | | | | | | | | | | | | |
| --- | --- | --- | --- | --- | --- | --- | --- | --- | --- | --- | --- | --- | --- | --- | --- | --- | --- | --- | --- | --- | --- | --- |
| Cluster | Rotation Function | | | | | Translation Function | | | | | Packing | | | | | Rigid Body Refinement | | | Initial CC | Best Trace CC/aa | | |
|  | #Rots. | Top LLG | Mean LLG | Top Zscore | Mean Zscore | #Trans. | Top LLG | Mean LLG | Top Zscore | Mean Zscore | #Sol. | Top LLG | Mean LLG | Top Zscore | Mean Zscore | #Sol. | Top LLG | Mean LLG | After Refinement CC | Cycle | CC | #Res. traced |
| 0 | 6 | 25.66 | 20.41 | 3.39 | 2.81 | 14 | 38.48 | 34.62 | 6.10 | 4.70 | 8 | 35.38 | 34.30 | 5.39 | 4.44 | 8 | 37.90 | 36.71 | 5.42 |  |  |  |
| 1 | 4 | 21.24 | 19.74 | 2.90 | 2.73 | 6 | 33.85 | 32.28 | 5.80 | 5.33 | 1 | 33.85 | 33.85 | 5.39 | 5.39 | 1 | 42.10 | 42.10 | 5.83 |  |  |  |
| 2 | 1 | 18.82 | 18.82 | 2.63 | 2.63 |  |  |  |  |  |  |  |  |  |  |  |  |  |  |  |  |  |
| 3 | 1 | 19.42 | 19.42 | 2.69 | 2.69 | 2 | 37.31 | 34.45 | 6.95 | 6.12 |  |  |  |  |  |  |  |  |  |  |  |  |
| 4 | 1 | 19.40 | 19.40 | 2.69 | 2.69 | 1 | 30.93 | 30.93 | 5.09 | 5.09 | 1 | 30.93 | 30.93 | 5.09 | 5.09 | 1 | 34.30 | 34.30 | 4.12 |  |  |  |

| Fragment 2 | | | | | | | | | | | | | | | | | | | | | | |
| --- | --- | --- | --- | --- | --- | --- | --- | --- | --- | --- | --- | --- | --- | --- | --- | --- | --- | --- | --- | --- | --- | --- |
| Cluster | Rotation Function | | | | | Translation Function | | | | | Packing | | | | | Rigid Body Refinement | | | Initial CC | Best Trace CC/aa | | |
|  | #Rots. | Top LLG | Mean LLG | Top Zscore | Mean Zscore | #Trans. | Top LLG | Mean LLG | Top Zscore | Mean Zscore | #Sol. | Top LLG | Mean LLG | Top Zscore | Mean Zscore | #Sol. | Top LLG | Mean LLG | After Refinement CC | Cycle | CC | #Res. traced |
| (0, 0) | 38 | 60.24 | 52.67 | 3.13 | 2.33 | 182 | 93.28 | 78.62 | 8.98 | 6.74 |  |  |  |  |  |  |  |  |  |  |  |  |
| (0, 6) | 16 | 57.70 | 54.74 | 2.76 | 2.57 | 111 | 92.65 | 78.75 | 8.92 | 6.70 |  |  |  |  |  |  |  |  |  |  |  |  |
| (0, 7) | 25 | 57.13 | 54.40 | 2.71 | 2.54 | 114 | 105.54 | 78.37 | 9.15 | 6.60 | 2 | 105.54 | 96.59 | 9.15 | 8.41 | 2 | 114.20 | 104.40 | 8.10 |  |  |  |
| (0, 8) | 23 | 57.87 | 54.80 | 2.81 | 2.58 | 108 | 115.27 | 80.54 | 11.25 | 7.28 | 1 | 115.27 | 115.27 | 11.25 | 11.25 | 1 | 128.10 | 128.10 | 9.88 |  |  |  |
| (0, 9) | 6 | 51.87 | 50.33 | 2.13 | 2.10 | 8 | 79.29 | 76.71 | 7.43 | 6.63 |  |  |  |  |  |  |  |  |  |  |  |  |
| (0, 10) | 8 | 57.07 | 55.64 | 2.76 | 2.67 | 54 | 87.81 | 78.39 | 8.34 | 6.48 |  |  |  |  |  |  |  |  |  |  |  |  |
| (0, 11) | 23 | 56.12 | 53.52 | 2.57 | 2.40 | 70 | 121.10 | 81.36 | 10.64 | 6.90 |  |  |  |  |  |  |  |  |  |  |  |  |
| (0, 12) | 2 | 54.54 | 52.81 | 2.39 | 2.38 | 3 | 79.23 | 76.67 | 7.21 | 6.49 |  |  |  |  |  |  |  |  |  |  |  |  |
| (0, 13) | 2 | 53.98 | 52.33 | 2.32 | 2.32 | 3 | 82.89 | 78.27 | 8.11 | 7.24 |  |  |  |  |  |  |  |  |  |  |  |  |
| (0, 14) | 23 | 56.79 | 52.96 | 2.73 | 2.37 | 69 | 119.06 | 83.72 | 10.59 | 7.05 |  |  |  |  |  |  |  |  |  |  |  |  |
| (0, 15) | 6 | 52.46 | 50.78 | 2.16 | 2.15 | 9 | 91.39 | 78.22 | 9.04 | 7.17 |  |  |  |  |  |  |  |  |  |  |  |  |
| (1, 5) | 7 | 62.63 | 57.98 | 2.91 | 2.37 | 15 | 103.83 | 89.66 | 8.86 | 7.33 |  |  |  |  |  |  |  |  |  |  |  |  |
| (1, 6) | 3 | 61.07 | 59.45 | 2.73 | 2.54 | 49 | 88.43 | 79.44 | 8.02 | 6.36 |  |  |  |  |  |  |  |  |  |  |  |  |
| (1, 7) | 7 | 60.88 | 58.94 | 2.71 | 2.48 | 44 | 95.03 | 82.97 | 8.07 | 6.59 |  |  |  |  |  |  |  |  |  |  |  |  |
| (1, 8) | 2 | 60.58 | 59.58 | 2.67 | 2.55 | 7 | 98.62 | 86.11 | 8.39 | 7.19 |  |  |  |  |  |  |  |  |  |  |  |  |
| (1, 9) | 1 | 60.37 | 60.37 | 2.65 | 2.65 | 25 | 83.88 | 78.00 | 7.35 | 5.80 |  |  |  |  |  |  |  |  |  |  |  |  |
| (1, 10) | 2 | 60.25 | 59.53 | 2.63 | 2.55 | 27 | 92.77 | 80.77 | 7.15 | 6.09 |  |  |  |  |  |  |  |  |  |  |  |  |
| (1, 11) | 3 | 59.29 | 58.22 | 2.52 | 2.40 | 39 | 93.19 | 81.74 | 8.55 | 6.60 |  |  |  |  |  |  |  |  |  |  |  |  |
| (1, 12) | 1 | 58.49 | 58.49 | 2.43 | 2.43 | 17 | 88.58 | 79.00 | 8.57 | 6.45 |  |  |  |  |  |  |  |  |  |  |  |  |
| (1, 13) | 1 | 57.69 | 57.69 | 2.34 | 2.34 | 2 | 99.81 | 91.06 | 8.16 | 7.52 |  |  |  |  |  |  |  |  |  |  |  |  |
| (4, 5) | 6 | 59.22 | 53.91 | 3.46 | 2.85 | 15 | 98.06 | 84.35 | 8.88 | 7.21 |  |  |  |  |  |  |  |  |  |  |  |  |
| (4, 10) | 1 | 52.73 | 52.73 | 2.71 | 2.71 | 7 | 85.43 | 78.35 | 8.22 | 7.12 |  |  |  |  |  |  |  |  |  |  |  |  |
| (4, 14) | 3 | 55.08 | 53.69 | 2.98 | 2.82 | 21 | 91.20 | 78.96 | 8.78 | 6.72 |  |  |  |  |  |  |  |  |  |  |  |  |
| (4, 16) | 1 | 52.24 | 52.24 | 2.65 | 2.65 | 8 | 88.39 | 78.40 | 8.62 | 7.21 |  |  |  |  |  |  |  |  |  |  |  |  |

| Fragment 3 | | | | | | | | | | | | | | | | | | | | | | |
| --- | --- | --- | --- | --- | --- | --- | --- | --- | --- | --- | --- | --- | --- | --- | --- | --- | --- | --- | --- | --- | --- | --- |
| Cluster | Rotation Function | | | | | Translation Function | | | | | Packing | | | | | Rigid Body Refinement | | | Initial CC | Best Trace CC/aa | | |
|  | #Rots. | Top LLG | Mean LLG | Top Zscore | Mean Zscore | #Trans. | Top LLG | Mean LLG | Top Zscore | Mean Zscore | #Sol. | Top LLG | Mean LLG | Top Zscore | Mean Zscore | #Sol. | Top LLG | Mean LLG | After Refinement CC | Cycle | CC | #Res. traced |
| (0, 0, 7) | 10 | 129.04 | 119.16 | 2.64 | 2.29 | 8 | 175.09 | 157.32 | 8.65 | 7.27 | 1 | 175.09 | 175.09 | 8.65 | 8.65 | 1 | 198.80 | 198.80 | 10.19 |  |  |  |
| (0, 0, 8) | 4 | 143.92 | 141.84 | 2.51 | 2.27 | 4 | 190.81 | 177.75 | 8.86 | 7.62 | 1 | 186.28 | 186.28 | 8.21 | 8.21 | 1 | 217.30 | 217.30 | 11.19 |  |  |  |
| (0, 7, 7) | 2 | 127.51 | 127.34 | 2.37 | 2.35 | 15 | 150.73 | 141.96 | 7.07 | 6.18 |  |  |  |  |  |  |  |  |  |  |  |  |
| (0, 7, 13) | 3 | 130.33 | 123.80 | 2.83 | 2.68 | 22 | 147.37 | 140.94 | 6.64 | 5.96 |  |  |  |  |  |  |  |  |  |  |  |  |
| (0, 7, 14) | 3 | 128.31 | 120.99 | 2.47 | 2.35 | 9 | 145.11 | 139.37 | 6.67 | 6.11 |  |  |  |  |  |  |  |  |  |  |  |  |
| (0, 7, 15) | 5 | 128.14 | 119.30 | 2.56 | 2.30 | 2 | 201.39 | 178.02 | 12.16 | 10.13 | 1 | 201.39 | 201.39 | 12.16 | 12.16 | 1 | 236.10 | 236.10 | 11.49 |  |  |  |
| (0, 7, 17) | 7 | 129.11 | 127.02 | 2.56 | 2.32 | 5 | 224.01 | 205.49 | 13.30 | 11.40 | 1 | 180.87 | 180.87 | 9.17 | 9.17 | 1 | 276.40 | 276.40 | 12.63 |  |  |  |
| (0, 7, 18) | 7 | 127.79 | 111.65 | 2.51 | 2.42 | 6 | 226.71 | 166.67 | 13.77 | 9.69 |  |  |  |  |  |  |  |  |  |  |  |  |
| (0, 7, 19) | 2 | 125.28 | 124.91 | 2.12 | 2.08 | 4 | 142.94 | 139.04 | 6.37 | 6.20 |  |  |  |  |  |  |  |  |  |  |  |  |
| (0, 7, 20) | 1 | 128.09 | 128.09 | 2.44 | 2.44 | 4 | 139.59 | 139.23 | 6.60 | 5.91 |  |  |  |  |  |  |  |  |  |  |  |  |
| (0, 7, 21) | 1 | 127.51 | 127.51 | 2.37 | 2.37 | 7 | 152.44 | 140.37 | 7.16 | 6.07 |  |  |  |  |  |  |  |  |  |  |  |  |
| (0, 7, 22) | 1 | 107.33 | 107.33 | 2.24 | 2.24 |  |  |  |  |  |  |  |  |  |  |  |  |  |  |  |  |  |
| (0, 8, 13) | 1 | 146.21 | 146.21 | 2.76 | 2.76 | 39 | 160.21 | 149.95 | 7.23 | 5.53 |  |  |  |  |  |  |  |  |  |  |  |  |
| (0, 8, 14) | 3 | 146.16 | 144.13 | 2.76 | 2.53 | 35 | 171.44 | 153.88 | 7.19 | 5.79 |  |  |  |  |  |  |  |  |  |  |  |  |
| (0, 8, 15) | 2 | 144.98 | 144.03 | 2.62 | 2.52 | 50 | 156.45 | 150.10 | 7.57 | 5.68 |  |  |  |  |  |  |  |  |  |  |  |  |
| (0, 8, 16) | 4 | 144.55 | 143.51 | 2.58 | 2.46 | 5 | 185.95 | 174.73 | 8.15 | 7.46 | 2 | 185.95 | 183.59 | 8.15 | 7.99 | 2 | 236.10 | 212.85 | 11.58 |  |  |  |
| (0, 8, 17) | 6 | 144.47 | 142.67 | 2.57 | 2.37 | 4 | 248.49 | 229.86 | 13.71 | 12.10 | 1 | 195.81 | 195.81 | 9.06 | 9.06 | 1 | 293.00 | 293.00 | 13.42 |  |  |  |
| (0, 8, 18) | 1 | 143.29 | 143.29 | 2.44 | 2.44 | 1 | 249.39 | 249.39 | 13.88 | 13.88 |  |  |  |  |  |  |  |  |  |  |  |  |
| (0, 8, 19) | 2 | 140.26 | 140.13 | 2.10 | 2.08 | 36 | 165.56 | 151.76 | 6.95 | 5.95 |  |  |  |  |  |  |  |  |  |  |  |  |

| Fragment 4 | | | | | | | | | | | | | | | | | | | | | | |
| --- | --- | --- | --- | --- | --- | --- | --- | --- | --- | --- | --- | --- | --- | --- | --- | --- | --- | --- | --- | --- | --- | --- |
| Cluster | Rotation Function | | | | | Translation Function | | | | | Packing | | | | | Rigid Body Refinement | | | Initial CC | Best Trace CC/aa | | |
|  | #Rots. | Top LLG | Mean LLG | Top Zscore | Mean Zscore | #Trans. | Top LLG | Mean LLG | Top Zscore | Mean Zscore | #Sol. | Top LLG | Mean LLG | Top Zscore | Mean Zscore | #Sol. | Top LLG | Mean LLG | After Refinement CC | Cycle | CC | #Res. traced |
| (0, 0, 0, 7) | 2 | 190.85 | 190.61 | 2.41 | 2.38 |  |  |  |  |  |  |  |  |  |  |  |  |  |  |  |  |  |
| (0, 0, 0, 8) | 1 | 203.00 | 203.00 | 2.37 | 2.37 |  |  |  |  |  |  |  |  |  |  |  |  |  |  |  |  |  |
| (0, 0, 7, 7) | 4 | 191.68 | 190.89 | 2.50 | 2.41 |  |  |  |  |  |  |  |  |  |  |  |  |  |  |  |  |  |
| (0, 0, 7, 15) | 3 | 246.58 | 245.67 | 2.41 | 2.31 | 3 | 307.01 | 289.63 | 9.93 | 9.34 |  |  |  |  |  |  |  |  |  |  |  |  |
| (0, 0, 7, 17) | 1 | 281.20 | 281.20 | 2.11 | 2.11 | 11 | 299.38 | 284.24 | 8.34 | 6.43 |  |  |  |  |  |  |  |  |  |  |  |  |
| (0, 0, 7, 46) | 2 | 193.00 | 192.04 | 2.65 | 2.54 |  |  |  |  |  |  |  |  |  |  |  |  |  |  |  |  |  |
| (0, 0, 7, 47) | 3 | 192.27 | 190.36 | 2.57 | 2.35 |  |  |  |  |  |  |  |  |  |  |  |  |  |  |  |  |  |
| (0, 0, 7, 49) | 3 | 191.45 | 189.88 | 2.48 | 2.30 | 1 | 275.02 | 275.02 | 13.01 | 13.01 | 1 | 275.02 | 275.02 | 13.01 | 13.01 | 1 | 342.90 | 342.90 | 13.47 |  |  |  |
| (0, 0, 7, 50) | 2 | 191.59 | 189.67 | 2.49 | 2.27 |  |  |  |  |  |  |  |  |  |  |  |  |  |  |  |  |  |
| (0, 0, 7, 51) | 1 | 191.36 | 191.36 | 2.47 | 2.47 |  |  |  |  |  |  |  |  |  |  |  |  |  |  |  |  |  |
| (0, 0, 7, 52) | 4 | 191.60 | 190.32 | 2.49 | 2.35 |  |  |  |  |  |  |  |  |  |  |  |  |  |  |  |  |  |
| (0, 0, 7, 53) | 4 | 193.71 | 190.58 | 2.73 | 2.38 |  |  |  |  |  |  |  |  |  |  |  |  |  |  |  |  |  |
| (0, 0, 7, 54) | 1 | 192.37 | 192.37 | 2.58 | 2.58 |  |  |  |  |  |  |  |  |  |  |  |  |  |  |  |  |  |
| (0, 0, 7, 55) | 2 | 191.50 | 190.64 | 2.48 | 2.38 |  |  |  |  |  |  |  |  |  |  |  |  |  |  |  |  |  |
| (0, 0, 7, 56) | 3 | 191.76 | 190.43 | 2.51 | 2.36 |  |  |  |  |  |  |  |  |  |  |  |  |  |  |  |  |  |
| (0, 0, 8, 16) | 10 | 241.18 | 215.44 | 2.46 | 2.23 | 9 | 297.18 | 263.31 | 9.66 | 9.07 | 4 | 297.18 | 270.31 | 9.66 | 9.06 | 3 | 339.20 | 307.27 | 12.12 |  |  |  |
| (0, 0, 8, 46) | 1 | 206.11 | 206.11 | 2.72 | 2.72 |  |  |  |  |  |  |  |  |  |  |  |  |  |  |  |  |  |
| (0, 0, 8, 47) | 3 | 206.81 | 205.01 | 2.79 | 2.59 |  |  |  |  |  |  |  |  |  |  |  |  |  |  |  |  |  |
| (0, 0, 8, 48) | 6 | 205.80 | 204.44 | 2.68 | 2.53 | 3 | 292.57 | 265.63 | 12.14 | 10.08 | 3 | 292.57 | 265.63 | 12.14 | 10.08 | 3 | 342.90 | 323.80 | 13.46 | 2 | 36.55 | 183 |
| (0, 0, 8, 49) | 2 | 205.85 | 205.38 | 2.69 | 2.63 |  |  |  |  |  |  |  |  |  |  |  |  |  |  |  |  |  |
| (0, 0, 8, 50) | 1 | 204.37 | 204.37 | 2.52 | 2.52 |  |  |  |  |  |  |  |  |  |  |  |  |  |  |  |  |  |
| (0, 0, 8, 52) | 4 | 203.45 | 202.62 | 2.42 | 2.33 | 1 | 251.52 | 251.52 | 9.66 | 9.66 |  |  |  |  |  |  |  |  |  |  |  |  |
| (0, 0, 8, 53) | 2 | 202.20 | 201.91 | 2.28 | 2.25 |  |  |  |  |  |  |  |  |  |  |  |  |  |  |  |  |  |
| (0, 7, 7, 15) | 2 | 246.47 | 246.26 | 2.39 | 2.37 | 50 | 254.74 | 243.57 | 6.42 | 5.60 |  |  |  |  |  |  |  |  |  |  |  |  |
| (0, 7, 7, 17) | 4 | 284.46 | 283.62 | 2.49 | 2.39 | 50 | 300.98 | 286.22 | 6.96 | 5.93 |  |  |  |  |  |  |  |  |  |  |  |  |
| (0, 7, 15, 46) | 1 | 250.16 | 250.16 | 2.81 | 2.81 | 50 | 254.97 | 242.44 | 6.78 | 5.31 |  |  |  |  |  |  |  |  |  |  |  |  |
| (0, 7, 15, 47) | 2 | 247.10 | 245.86 | 2.47 | 2.33 | 32 | 257.83 | 241.95 | 7.16 | 5.78 |  |  |  |  |  |  |  |  |  |  |  |  |
| (0, 7, 15, 49) | 2 | 245.88 | 244.91 | 2.33 | 2.22 | 50 | 253.96 | 241.31 | 6.81 | 5.68 |  |  |  |  |  |  |  |  |  |  |  |  |
| (0, 7, 15, 50) | 1 | 247.66 | 247.66 | 2.53 | 2.53 | 39 | 252.40 | 240.77 | 7.09 | 5.38 |  |  |  |  |  |  |  |  |  |  |  |  |
| (0, 7, 15, 52) | 5 | 247.92 | 246.66 | 2.56 | 2.42 | 5 | 381.68 | 341.67 | 15.90 | 12.79 | 2 | 316.34 | 294.26 | 10.75 | 9.38 | 2 | 448.80 | 369.15 | 16.03 |  |  |  |
| (0, 7, 15, 53) | 1 | 244.18 | 244.18 | 2.14 | 2.14 | 32 | 254.02 | 240.54 | 6.91 | 5.58 |  |  |  |  |  |  |  |  |  |  |  |  |
| (0, 7, 15, 57) | 1 | 246.17 | 246.17 | 2.36 | 2.36 | 1 | 384.09 | 384.09 | 16.01 | 16.01 |  |  |  |  |  |  |  |  |  |  |  |  |
| (0, 7, 17, 46) | 4 | 287.05 | 284.16 | 2.78 | 2.45 | 18 | 310.24 | 291.77 | 7.63 | 6.49 |  |  |  |  |  |  |  |  |  |  |  |  |
| (0, 7, 17, 47) | 2 | 284.56 | 283.44 | 2.50 | 2.37 | 35 | 297.86 | 281.72 | 7.11 | 5.84 |  |  |  |  |  |  |  |  |  |  |  |  |
| (0, 7, 17, 49) | 3 | 283.75 | 282.22 | 2.40 | 2.23 | 1 | 409.91 | 409.91 | 15.90 | 15.90 | 1 | 409.91 | 409.91 | 15.90 | 15.90 | 1 | 448.90 | 448.90 | 16.04 |  |  |  |
| (0, 7, 17, 50) | 2 | 286.54 | 285.64 | 2.73 | 2.62 | 50 | 293.89 | 281.55 | 7.41 | 5.55 |  |  |  |  |  |  |  |  |  |  |  |  |
| (0, 7, 17, 51) | 2 | 284.79 | 283.29 | 2.52 | 2.35 | 37 | 303.17 | 285.15 | 6.95 | 5.91 |  |  |  |  |  |  |  |  |  |  |  |  |
| (0, 7, 17, 52) | 1 | 283.18 | 283.18 | 2.34 | 2.34 | 38 | 293.81 | 278.02 | 6.94 | 5.56 |  |  |  |  |  |  |  |  |  |  |  |  |
| (0, 7, 17, 53) | 2 | 286.68 | 284.58 | 2.74 | 2.50 | 50 | 296.66 | 282.36 | 6.64 | 5.57 |  |  |  |  |  |  |  |  |  |  |  |  |
| (0, 7, 17, 54) | 2 | 286.16 | 284.74 | 2.68 | 2.52 | 27 | 303.87 | 286.23 | 7.31 | 5.80 |  |  |  |  |  |  |  |  |  |  |  |  |
| (0, 7, 17, 55) | 2 | 285.56 | 284.37 | 2.61 | 2.47 | 6 | 315.72 | 296.13 | 8.59 | 6.76 |  |  |  |  |  |  |  |  |  |  |  |  |
| (0, 7, 17, 56) | 2 | 284.63 | 284.29 | 2.51 | 2.47 | 50 | 295.68 | 281.94 | 6.94 | 5.59 |  |  |  |  |  |  |  |  |  |  |  |  |
| (0, 8, 16, 16) | 2 | 241.15 | 241.11 | 2.37 | 2.37 | 32 | 259.83 | 240.87 | 7.14 | 5.80 |  |  |  |  |  |  |  |  |  |  |  |  |
| (0, 8, 16, 46) | 2 | 244.38 | 224.03 | 2.74 | 2.74 | 24 | 248.02 | 239.58 | 6.85 | 5.57 |  |  |  |  |  |  |  |  |  |  |  |  |
| (0, 8, 16, 47) | 3 | 242.13 | 227.11 | 2.48 | 2.32 | 28 | 250.14 | 239.29 | 7.30 | 5.78 |  |  |  |  |  |  |  |  |  |  |  |  |
| (0, 8, 16, 49) | 3 | 241.14 | 214.17 | 2.55 | 2.39 | 24 | 245.21 | 237.28 | 6.68 | 5.75 |  |  |  |  |  |  |  |  |  |  |  |  |
| (0, 8, 16, 50) | 1 | 242.29 | 242.29 | 2.50 | 2.50 | 18 | 250.30 | 239.04 | 6.85 | 5.65 |  |  |  |  |  |  |  |  |  |  |  |  |
| (0, 8, 16, 52) | 10 | 242.67 | 220.57 | 2.54 | 2.35 | 8 | 375.92 | 323.95 | 15.99 | 13.85 |  |  |  |  |  |  |  |  |  |  |  |  |
| (0, 8, 16, 53) | 3 | 239.31 | 225.20 | 2.17 | 2.11 | 29 | 251.35 | 239.21 | 7.06 | 5.97 |  |  |  |  |  |  |  |  |  |  |  |  |
| (0, 8, 16, 57) | 1 | 241.34 | 241.34 | 2.39 | 2.39 | 1 | 383.51 | 383.51 | 16.32 | 16.32 |  |  |  |  |  |  |  |  |  |  |  |  |
| (0, 8, 17, 46) | 2 | 309.89 | 308.57 | 2.88 | 2.73 | 12 | 332.27 | 313.21 | 7.49 | 6.17 |  |  |  |  |  |  |  |  |  |  |  |  |
| (0, 8, 17, 47) | 3 | 308.91 | 306.95 | 2.77 | 2.55 | 24 | 330.69 | 311.95 | 7.17 | 6.05 |  |  |  |  |  |  |  |  |  |  |  |  |
| (0, 8, 17, 48) | 7 | 308.78 | 306.95 | 2.76 | 2.55 | 5 | 416.74 | 362.82 | 13.82 | 9.91 | 5 | 416.74 | 362.82 | 13.82 | 9.91 | 5 | 450.10 | 409.92 | 16.03 |  |  |  |
| (0, 8, 17, 49) | 2 | 307.29 | 306.56 | 2.59 | 2.50 | 50 | 311.25 | 302.42 | 6.32 | 5.51 |  |  |  |  |  |  |  |  |  |  |  |  |
| (0, 8, 17, 50) | 1 | 306.01 | 306.01 | 2.45 | 2.45 | 28 | 315.40 | 302.14 | 7.44 | 5.58 |  |  |  |  |  |  |  |  |  |  |  |  |
| (0, 8, 17, 51) | 1 | 305.64 | 305.64 | 2.40 | 2.40 | 7 | 327.21 | 310.60 | 7.27 | 6.40 |  |  |  |  |  |  |  |  |  |  |  |  |
| (0, 8, 17, 52) | 1 | 305.11 | 305.11 | 2.34 | 2.34 | 9 | 324.83 | 308.06 | 7.36 | 6.24 |  |  |  |  |  |  |  |  |  |  |  |  |
| (0, 8, 17, 53) | 1 | 303.72 | 303.72 | 2.19 | 2.19 | 20 | 320.92 | 305.91 | 7.02 | 5.84 |  |  |  |  |  |  |  |  |  |  |  |  |

| Fragment 5 | | | | | | | | | | | | | | | | | | | | | | |
| --- | --- | --- | --- | --- | --- | --- | --- | --- | --- | --- | --- | --- | --- | --- | --- | --- | --- | --- | --- | --- | --- | --- |
| Cluster | Rotation Function | | | | | Translation Function | | | | | Packing | | | | | Rigid Body Refinement | | | Initial CC | Best Trace CC/aa | | |
|  | #Rots. | Top LLG | Mean LLG | Top Zscore | Mean Zscore | #Trans. | Top LLG | Mean LLG | Top Zscore | Mean Zscore | #Sol. | Top LLG | Mean LLG | Top Zscore | Mean Zscore | #Sol. | Top LLG | Mean LLG | After Refinement CC | Cycle | CC | #Res. traced |
| (0, 0, 0, 7, 49) | 2 | 320.51 | 320.10 | 2.35 | 2.30 |  |  |  |  |  |  |  |  |  |  |  |  |  |  |  |  |  |
| (0, 0, 0, 8, 16) | 4 | 317.17 | 294.68 | 2.42 | 2.34 |  |  |  |  |  |  |  |  |  |  |  |  |  |  |  |  |  |
| (0, 0, 0, 8, 48) | 6 | 321.12 | 301.46 | 2.44 | 2.33 |  |  |  |  |  |  |  |  |  |  |  |  |  |  |  |  |  |
| (0, 0, 7, 7, 49) | 6 | 322.33 | 321.07 | 2.55 | 2.41 |  |  |  |  |  |  |  |  |  |  |  |  |  |  |  |  |  |
| (0, 0, 7, 15, 52) | 1 | 292.47 | 292.47 | 2.29 | 2.29 |  |  |  |  |  |  |  |  |  |  |  |  |  |  |  |  |  |
| (0, 0, 7, 49, 116) | 2 | 323.98 | 322.47 | 2.73 | 2.56 |  |  |  |  |  |  |  |  |  |  |  |  |  |  |  |  |  |
| (0, 0, 7, 49, 117) | 2 | 324.44 | 323.69 | 2.78 | 2.70 |  |  |  |  |  |  |  |  |  |  |  |  |  |  |  |  |  |
| (0, 0, 7, 49, 118) | 4 | 323.57 | 321.11 | 2.69 | 2.42 |  |  |  |  |  |  |  |  |  |  |  |  |  |  |  |  |  |
| (0, 0, 7, 49, 121) | 3 | 322.86 | 321.16 | 2.61 | 2.42 |  |  |  |  |  |  |  |  |  |  |  |  |  |  |  |  |  |
| (0, 0, 7, 49, 122) | 2 | 321.42 | 320.06 | 2.45 | 2.30 |  |  |  |  |  |  |  |  |  |  |  |  |  |  |  |  |  |
| (0, 0, 7, 49, 123) | 3 | 322.34 | 321.07 | 2.55 | 2.41 |  |  |  |  |  |  |  |  |  |  |  |  |  |  |  |  |  |
| (0, 0, 7, 49, 124) | 3 | 321.58 | 320.81 | 2.47 | 2.38 | 1 | 390.25 | 390.25 | 11.08 | 11.08 |  |  |  |  |  |  |  |  |  |  |  |  |
| (0, 0, 7, 49, 126) | 2 | 320.82 | 320.31 | 2.38 | 2.33 |  |  |  |  |  |  |  |  |  |  |  |  |  |  |  |  |  |
| (0, 0, 8, 16, 16) | 9 | 318.90 | 304.69 | 2.55 | 2.38 |  |  |  |  |  |  |  |  |  |  |  |  |  |  |  |  |  |
| (0, 0, 8, 16, 116) | 5 | 319.92 | 298.97 | 2.75 | 2.59 |  |  |  |  |  |  |  |  |  |  |  |  |  |  |  |  |  |
| (0, 0, 8, 16, 117) | 5 | 321.15 | 299.50 | 2.88 | 2.65 |  |  |  |  |  |  |  |  |  |  |  |  |  |  |  |  |  |
| (0, 0, 8, 16, 118) | 8 | 317.72 | 291.82 | 2.87 | 2.37 |  |  |  |  |  |  |  |  |  |  |  |  |  |  |  |  |  |
| (0, 0, 8, 16, 121) | 4 | 319.27 | 305.95 | 2.59 | 2.35 |  |  |  |  |  |  |  |  |  |  |  |  |  |  |  |  |  |
| (0, 0, 8, 16, 122) | 3 | 318.33 | 293.56 | 2.49 | 2.45 |  |  |  |  |  |  |  |  |  |  |  |  |  |  |  |  |  |
| (0, 0, 8, 16, 123) | 5 | 319.04 | 296.97 | 2.56 | 2.37 |  |  |  |  |  |  |  |  |  |  |  |  |  |  |  |  |  |
| (0, 0, 8, 16, 124) | 10 | 317.91 | 290.39 | 2.49 | 2.32 | 2 | 383.86 | 376.78 | 12.01 | 11.44 |  |  |  |  |  |  |  |  |  |  |  |  |
| (0, 0, 8, 16, 126) | 5 | 317.83 | 293.89 | 2.63 | 2.39 |  |  |  |  |  |  |  |  |  |  |  |  |  |  |  |  |  |
| (0, 0, 8, 16, 128) | 1 | 269.71 | 269.71 | 2.04 | 2.04 |  |  |  |  |  |  |  |  |  |  |  |  |  |  |  |  |  |
| (0, 0, 8, 48, 48) | 10 | 322.84 | 318.43 | 2.55 | 2.40 |  |  |  |  |  |  |  |  |  |  |  |  |  |  |  |  |  |
| (0, 0, 8, 48, 116) | 5 | 324.60 | 310.18 | 2.73 | 2.58 |  |  |  |  |  |  |  |  |  |  |  |  |  |  |  |  |  |
| (0, 0, 8, 48, 117) | 5 | 325.11 | 310.99 | 2.81 | 2.67 |  |  |  |  |  |  |  |  |  |  |  |  |  |  |  |  |  |
| (0, 0, 8, 48, 118) | 7 | 321.22 | 303.96 | 2.41 | 2.29 |  |  |  |  |  |  |  |  |  |  |  |  |  |  |  |  |  |
| (0, 0, 8, 48, 121) | 8 | 323.45 | 306.10 | 2.60 | 2.39 |  |  |  |  |  |  |  |  |  |  |  |  |  |  |  |  |  |
| (0, 0, 8, 48, 122) | 4 | 322.03 | 306.74 | 2.48 | 2.37 |  |  |  |  |  |  |  |  |  |  |  |  |  |  |  |  |  |
| (0, 0, 8, 48, 123) | 6 | 322.95 | 310.78 | 2.56 | 2.40 |  |  |  |  |  |  |  |  |  |  |  |  |  |  |  |  |  |
| (0, 0, 8, 48, 124) | 9 | 322.09 | 301.59 | 2.45 | 2.34 | 2 | 390.06 | 385.48 | 11.19 | 11.10 |  |  |  |  |  |  |  |  |  |  |  |  |
| (0, 0, 8, 48, 126) | 6 | 321.47 | 302.01 | 2.62 | 2.39 |  |  |  |  |  |  |  |  |  |  |  |  |  |  |  |  |  |
| (0, 7, 7, 15, 52) | 6 | 454.71 | 426.74 | 2.52 | 2.38 | 26 | 474.06 | 451.57 | 7.84 | 6.35 |  |  |  |  |  |  |  |  |  |  |  |  |
| (0, 7, 7, 17, 49) | 4 | 454.14 | 453.17 | 2.50 | 2.40 | 50 | 462.81 | 443.67 | 6.79 | 5.93 |  |  |  |  |  |  |  |  |  |  |  |  |
| (0, 7, 15, 52, 116) | 3 | 458.06 | 403.70 | 2.92 | 2.78 | 22 | 469.13 | 448.22 | 7.38 | 6.00 |  |  |  |  |  |  |  |  |  |  |  |  |
| (0, 7, 15, 52, 117) | 3 | 457.25 | 402.52 | 2.80 | 2.65 | 24 | 466.02 | 446.45 | 7.46 | 5.98 |  |  |  |  |  |  |  |  |  |  |  |  |
| (0, 7, 15, 52, 118) | 3 | 456.46 | 453.54 | 2.71 | 2.39 | 50 | 455.66 | 443.17 | 6.91 | 5.83 |  |  |  |  |  |  |  |  |  |  |  |  |
| (0, 7, 15, 52, 119) | 1 | 453.35 | 453.35 | 2.37 | 2.37 | 6 | 477.35 | 453.54 | 8.12 | 6.49 |  |  |  |  |  |  |  |  |  |  |  |  |
| (0, 7, 15, 52, 120) | 2 | 455.12 | 454.12 | 2.56 | 2.45 | 50 | 459.60 | 442.67 | 7.16 | 5.74 |  |  |  |  |  |  |  |  |  |  |  |  |
| (0, 7, 15, 52, 121) | 2 | 454.87 | 454.43 | 2.54 | 2.49 | 50 | 451.19 | 440.95 | 6.72 | 5.60 |  |  |  |  |  |  |  |  |  |  |  |  |
| (0, 7, 15, 52, 122) | 2 | 454.32 | 374.06 | 2.47 | 2.46 | 13 | 465.68 | 444.48 | 7.24 | 6.03 |  |  |  |  |  |  |  |  |  |  |  |  |
| (0, 7, 15, 52, 123) | 2 | 454.25 | 373.56 | 2.47 | 2.40 | 15 | 460.78 | 444.30 | 7.16 | 6.05 |  |  |  |  |  |  |  |  |  |  |  |  |
| (0, 7, 15, 52, 124) | 1 | 453.44 | 453.44 | 2.38 | 2.38 | 50 | 450.38 | 435.47 | 6.49 | 5.51 |  |  |  |  |  |  |  |  |  |  |  |  |
| (0, 7, 15, 52, 126) | 2 | 452.77 | 372.36 | 2.30 | 2.26 | 23 | 456.16 | 438.06 | 7.54 | 5.79 |  |  |  |  |  |  |  |  |  |  |  |  |
| (0, 7, 15, 52, 127) | 3 | 292.40 | 292.22 | 2.28 | 2.26 | 3 | 407.07 | 399.88 | 14.27 | 13.83 |  |  |  |  |  |  |  |  |  |  |  |  |
| (0, 7, 17, 49, 116) | 3 | 457.60 | 455.08 | 2.89 | 2.61 | 42 | 466.41 | 445.44 | 7.23 | 5.93 |  |  |  |  |  |  |  |  |  |  |  |  |
| (0, 7, 17, 49, 117) | 2 | 456.71 | 455.66 | 2.79 | 2.67 | 39 | 462.58 | 442.63 | 7.40 | 5.74 |  |  |  |  |  |  |  |  |  |  |  |  |
| (0, 7, 17, 49, 118) | 3 | 455.89 | 453.03 | 2.70 | 2.38 | 50 | 455.18 | 442.72 | 7.01 | 5.85 |  |  |  |  |  |  |  |  |  |  |  |  |
| (0, 7, 17, 49, 119) | 1 | 452.87 | 452.87 | 2.36 | 2.36 | 3 | 478.11 | 458.72 | 8.66 | 6.99 |  |  |  |  |  |  |  |  |  |  |  |  |
| (0, 7, 17, 49, 120) | 2 | 454.63 | 453.63 | 2.56 | 2.45 | 50 | 459.02 | 442.19 | 7.16 | 5.78 |  |  |  |  |  |  |  |  |  |  |  |  |
| (0, 7, 17, 49, 121) | 2 | 454.41 | 453.95 | 2.53 | 2.48 | 50 | 450.53 | 440.00 | 6.68 | 5.60 |  |  |  |  |  |  |  |  |  |  |  |  |
| (0, 7, 17, 49, 122) | 1 | 453.77 | 453.77 | 2.46 | 2.46 | 15 | 463.83 | 443.09 | 7.15 | 5.91 |  |  |  |  |  |  |  |  |  |  |  |  |
| (0, 7, 17, 49, 123) | 1 | 453.67 | 453.67 | 2.45 | 2.45 | 16 | 459.42 | 443.05 | 7.10 | 6.01 |  |  |  |  |  |  |  |  |  |  |  |  |
| (0, 7, 17, 49, 124) | 1 | 453.03 | 453.03 | 2.38 | 2.38 | 50 | 450.61 | 435.31 | 6.57 | 5.50 |  |  |  |  |  |  |  |  |  |  |  |  |
| (0, 7, 17, 49, 126) | 1 | 452.41 | 452.41 | 2.31 | 2.31 | 20 | 457.03 | 438.93 | 7.61 | 5.83 |  |  |  |  |  |  |  |  |  |  |  |  |
| (0, 8, 17, 48, 48) | 15 | 457.86 | 451.76 | 2.54 | 2.38 | 87 | 479.46 | 442.97 | 8.15 | 6.26 |  |  |  |  |  |  |  |  |  |  |  |  |
| (0, 8, 17, 48, 116) | 14 | 460.90 | 416.48 | 2.88 | 2.64 | 143 | 470.12 | 413.80 | 7.49 | 5.90 |  |  |  |  |  |  |  |  |  |  |  |  |
| (0, 8, 17, 48, 117) | 8 | 460.14 | 431.87 | 2.79 | 2.61 | 114 | 471.70 | 427.98 | 8.04 | 5.86 | 2 | 382.60 | 372.81 | 8.04 | 7.97 | 2 | 498.40 | 486.55 | 15.93 | 3 | 36.5 | 182 |
| (0, 8, 17, 48, 118) | 6 | 459.28 | 455.02 | 2.70 | 2.38 | 100 | 459.13 | 444.68 | 6.96 | 5.88 |  |  |  |  |  |  |  |  |  |  |  |  |
| (0, 8, 17, 48, 119) | 8 | 456.39 | 418.91 | 2.60 | 2.43 | 42 | 481.45 | 404.21 | 8.79 | 6.45 | 1 | 468.46 | 468.46 | 7.64 | 7.64 | 1 | 504.80 | 504.80 | 15.75 |  |  |  |
| (0, 8, 17, 48, 120) | 2 | 457.67 | 456.32 | 2.52 | 2.52 | 60 | 460.45 | 442.88 | 7.19 | 5.68 |  |  |  |  |  |  |  |  |  |  |  |  |
| (0, 8, 17, 48, 121) | 9 | 457.94 | 434.07 | 2.56 | 2.43 | 200 | 455.27 | 419.31 | 6.86 | 5.60 |  |  |  |  |  |  |  |  |  |  |  |  |
| (0, 8, 17, 48, 122) | 7 | 457.21 | 426.21 | 2.58 | 2.41 | 104 | 465.27 | 429.97 | 7.22 | 5.90 |  |  |  |  |  |  |  |  |  |  |  |  |
| (0, 8, 17, 48, 123) | 8 | 457.36 | 429.07 | 2.49 | 2.30 | 134 | 465.57 | 421.31 | 7.25 | 5.77 |  |  |  |  |  |  |  |  |  |  |  |  |
| (0, 8, 17, 48, 124) | 5 | 456.23 | 412.34 | 2.45 | 2.34 | 145 | 453.69 | 431.64 | 7.05 | 5.51 |  |  |  |  |  |  |  |  |  |  |  |  |
| (0, 8, 17, 48, 125) | 8 | 452.97 | 396.63 | 2.38 | 2.28 | 118 | 456.85 | 400.94 | 7.23 | 5.74 |  |  |  |  |  |  |  |  |  |  |  |  |
| (0, 8, 17, 48, 126) | 8 | 454.98 | 396.33 | 2.55 | 2.36 | 124 | 458.53 | 404.38 | 7.53 | 5.69 |  |  |  |  |  |  |  |  |  |  |  |  |

| Fragment 6 | | | | | | | | | | | | | | | | | | | | | | |
| --- | --- | --- | --- | --- | --- | --- | --- | --- | --- | --- | --- | --- | --- | --- | --- | --- | --- | --- | --- | --- | --- | --- |
| Cluster | Rotation Function | | | | | Translation Function | | | | | Packing | | | | | Rigid Body Refinement | | | Initial CC | Best Trace CC/aa | | |
|  | #Rots. | Top LLG | Mean LLG | Top Zscore | Mean Zscore | #Trans. | Top LLG | Mean LLG | Top Zscore | Mean Zscore | #Sol. | Top LLG | Mean LLG | Top Zscore | Mean Zscore | #Sol. | Top LLG | Mean LLG | After Refinement CC | Cycle | CC | #Res. traced |
| (0, 0, 8, 17, 48, 117) | 2 | 502.28 | 490.77 | 2.26 | 2.24 | 4 | 515.37 | 492.55 | 8.76 | 7.44 |  |  |  |  |  |  |  |  |  |  |  |  |
| (0, 8, 17, 17, 48, 117) | 1 | 503.64 | 503.64 | 2.37 | 2.37 | 23 | 486.20 | 473.64 | 7.03 | 5.65 |  |  |  |  |  |  |  |  |  |  |  |  |
| (0, 8, 17, 48, 48, 117) | 2 | 502.33 | 490.66 | 2.23 | 2.23 | 16 | 481.64 | 472.80 | 6.21 | 5.50 |  |  |  |  |  |  |  |  |  |  |  |  |
| (0, 8, 17, 48, 48, 119) | 4 | 503.87 | 502.76 | 2.53 | 2.41 | 50 | 501.35 | 479.33 | 7.01 | 5.83 |  |  |  |  |  |  |  |  |  |  |  |  |
| (0, 8, 17, 48, 117, 117) | 5 | 503.48 | 493.52 | 2.35 | 2.29 | 50 | 492.66 | 475.94 | 6.71 | 5.60 |  |  |  |  |  |  |  |  |  |  |  |  |
| (0, 8, 17, 48, 117, 222) | 4 | 508.06 | 495.01 | 2.90 | 2.72 | 53 | 492.84 | 480.23 | 7.18 | 5.86 |  |  |  |  |  |  |  |  |  |  |  |  |
| (0, 8, 17, 48, 117, 223) | 2 | 503.87 | 492.32 | 2.43 | 2.42 | 26 | 489.93 | 475.65 | 6.73 | 5.58 |  |  |  |  |  |  |  |  |  |  |  |  |
| (0, 8, 17, 48, 117, 227) | 4 | 504.61 | 492.42 | 2.48 | 2.43 | 50 | 495.36 | 476.05 | 6.57 | 5.51 |  |  |  |  |  |  |  |  |  |  |  |  |
| (0, 8, 17, 48, 117, 228) | 4 | 504.76 | 491.77 | 2.52 | 2.36 | 52 | 492.54 | 477.29 | 6.95 | 5.75 |  |  |  |  |  |  |  |  |  |  |  |  |
| (0, 8, 17, 48, 117, 229) | 6 | 505.37 | 492.79 | 2.59 | 2.47 | 19 | 514.25 | 486.47 | 7.71 | 6.50 | 2 | 514.25 | 501.77 | 7.71 | 7.62 | 2 | 534.00 | 521.30 | 16.04 |  |  |  |
| (0, 8, 17, 48, 117, 230) | 2 | 481.05 | 480.87 | 2.46 | 2.44 |  |  |  |  |  |  |  |  |  |  |  |  |  |  |  |  |  |
| (0, 8, 17, 48, 117, 232) | 2 | 503.23 | 491.53 | 2.33 | 2.33 | 17 | 500.90 | 477.78 | 7.06 | 5.79 |  |  |  |  |  |  |  |  |  |  |  |  |
| (0, 8, 17, 48, 119, 222) | 3 | 506.29 | 504.49 | 2.81 | 2.60 | 50 | 496.22 | 476.03 | 7.05 | 5.58 |  |  |  |  |  |  |  |  |  |  |  |  |
| (0, 8, 17, 48, 119, 223) | 2 | 506.25 | 504.86 | 2.80 | 2.65 | 19 | 503.48 | 482.59 | 7.55 | 5.97 |  |  |  |  |  |  |  |  |  |  |  |  |
| (0, 8, 17, 48, 119, 224) | 2 | 504.68 | 503.43 | 2.62 | 2.49 | 25 | 499.69 | 480.57 | 7.29 | 5.79 |  |  |  |  |  |  |  |  |  |  |  |  |
| (0, 8, 17, 48, 119, 225) | 1 | 504.51 | 504.51 | 2.61 | 2.61 | 29 | 496.28 | 477.27 | 7.24 | 5.76 |  |  |  |  |  |  |  |  |  |  |  |  |
| (0, 8, 17, 48, 119, 226) | 1 | 503.89 | 503.89 | 2.54 | 2.54 | 29 | 492.98 | 475.50 | 6.86 | 5.56 |  |  |  |  |  |  |  |  |  |  |  |  |
| (0, 8, 17, 48, 119, 227) | 2 | 503.76 | 503.62 | 2.52 | 2.50 | 28 | 501.63 | 479.64 | 6.83 | 5.76 |  |  |  |  |  |  |  |  |  |  |  |  |
| (0, 8, 17, 48, 119, 228) | 3 | 503.22 | 501.48 | 2.46 | 2.27 | 42 | 495.12 | 476.39 | 6.87 | 5.68 |  |  |  |  |  |  |  |  |  |  |  |  |
| (0, 8, 17, 48, 119, 229) | 1 | 502.61 | 502.61 | 2.39 | 2.39 | 17 | 492.44 | 474.81 | 6.82 | 5.65 |  |  |  |  |  |  |  |  |  |  |  |  |
| (0, 8, 17, 48, 119, 230) | 2 | 502.42 | 501.31 | 2.37 | 2.25 | 32 | 488.40 | 476.42 | 6.91 | 5.90 |  |  |  |  |  |  |  |  |  |  |  |  |
| (0, 8, 17, 48, 119, 231) | 2 | 501.92 | 501.33 | 2.32 | 2.25 | 43 | 492.16 | 474.62 | 6.91 | 5.71 |  |  |  |  |  |  |  |  |  |  |  |  |
| (0, 8, 17, 48, 119, 232) | 1 | 500.93 | 500.93 | 2.20 | 2.20 | 15 | 493.74 | 477.41 | 7.05 | 5.88 |  |  |  |  |  |  |  |  |  |  |  |  |


The current best solution is: th10\_0\_0xx4FR3\_7-1.pdb with FINALCC: 36.55 and n. residues traced 183  
file is: gr/10\_6/8\_EXP\_LIBRARY/2/0/th10\_0\_0xx4FR3\_7-1.pdb

- FRF: Pos. in Rank: **107** LLG: **204.85** ZSCORE: **2.57** Top LLG in Cluster **(0, 0, 8, 48)**: **309.89** Top ZSCORE in Cluster **(0, 0, 8, 48)**: **2.88**
- REFINEMENT ROTATION AND MODEL

- FTF: Pos. in Rank: **556** LLG: **292.57** ZSCORE: **12.14** Top LLG in Cluster **(0, 0, 8, 48)**: **416.74** Top ZSCORE in Cluster **(0, 0, 8, 48)**: **16.32**
- PACK: Pos. in Rank: **13** LLG: **292.57** ZSCORE: **12.14** Top LLG in Cluster **(0, 0, 8, 48)**: **416.74** Top ZSCORE in Cluster **(0, 0, 8, 48)**: **15.90**
- RNP: Pos. in Rank: **9** LLG: **342.90** ZSCORE: **0.00** Top LLG in Cluster **(0, 0, 8, 48)**: **450.10** Top ZSCORE in Cluster **(0, 0, 8, 48)**: **0.00**
- INITIAL CC

- After Refinement: Pos. in Rank: **8** INITCC: **13.46** Top INITCC in Cluster **(0, 0, 8, 48)**: **16.04**

- EXPANSION

Cycle 2:: Final CC: **36.55%** N. Residues Traced: **183.00**

# It seems you have a good solution! Here you can find the best solution and map for further refinement.

Time MODE: ARCIMBOLDO-CLUSTERS STEP: FRF 2014-08-10 10:03  
Time MODE: ARCIMBOLDO STEP: TABLE 2014-08-10 10:03  
Time MODE: ARCIMBOLDO STEP: FTF 2014-08-10 10:04  
Time MODE: ARCIMBOLDO STEP: PACK 2014-08-10 10:04  
Time MODE: ARCIMBOLDO STEP: RNP 2014-08-10 10:04  
Time MODE: ARCIMBOLDO STEP: INITCC 2014-08-10 10:04  
Time MODE: ARCIMBOLDO STEP: TABLE 2014-08-10 10:05  
Time MODE: ARCIMBOLDO STEP: FTF 2014-08-10 10:06  
Time MODE: ARCIMBOLDO STEP: PACK 2014-08-10 10:06  
Time MODE: ARCIMBOLDO STEP: RNP 2014-08-10 10:06  
Time MODE: ARCIMBOLDO STEP: INITCC 2014-08-10 10:06  
Time MODE: ARCIMBOLDO STEP: TABLE 2014-08-10 10:06  
Time MODE: ARCIMBOLDO STEP: FTF 2014-08-10 10:07  
Time MODE: ARCIMBOLDO STEP: PACK 2014-08-10 10:07  
Time MODE: ARCIMBOLDO STEP: RNP 2014-08-10 10:07  
Time MODE: ARCIMBOLDO STEP: INITCC 2014-08-10 10:07  
Time MODE: ARCIMBOLDO STEP: TABLE 2014-08-10 10:07  
Time MODE: ARCIMBOLDO STEP: FTF 2014-08-10 10:09  
Time MODE: ARCIMBOLDO STEP: PACK 2014-08-10 10:09  
Time MODE: ARCIMBOLDO STEP: RNP 2014-08-10 10:09  
Time MODE: ARCIMBOLDO STEP: INITCC 2014-08-10 10:09  
Time MODE: ARCIMBOLDO STEP: TABLE 2014-08-10 10:10  
Time MODE: ARCIMBOLDO STEP: FTF 2014-08-10 10:12  
Time MODE: ARCIMBOLDO STEP: PACK 2014-08-10 10:12  
Time MODE: ARCIMBOLDO STEP: RNP 2014-08-10 10:13  
Time MODE: ARCIMBOLDO STEP: INITCC 2014-08-10 10:13  
Time MODE: ARCIMBOLDO STEP: TABLE 2014-08-10 10:13  
Time MODE: ARCIMBOLDO STEP: FTF 2014-08-10 10:14  
Time MODE: ARCIMBOLDO STEP: PACK 2014-08-10 10:14  
Time MODE: ARCIMBOLDO STEP: RNP 2014-08-10 10:14  
Time MODE: ARCIMBOLDO STEP: INITCC 2014-08-10 10:14  
Time MODE: ARCIMBOLDO STEP: FAST\_1 2014-08-10 10:47  
Time MODE: ARCIMBOLDO STEP: FAST\_2 2014-08-10 11:13  
Time MODE: ARCIMBOLDO STEP: FAST\_3 2014-08-10 11:40

In case this result is helpful, please, cite   

Sammito, M., Millán, C., Rodríguez, D. D., M. de Ilarduya, I., Meindl, K., De Marino, I., Petrillo, G., Buey, R. M., de Pereda, J. M., Zeth, K., Sheldrick, G. M. & Usón, I.   
 "Exploiting tertiary structure through local folds for crystallographic phasing"   
 Nat Methods. 10, 1099-1101. Nature Methods (2013) doi:10.1038/nmeth.2644

Crystallographic Methods Group.   
 Department Structural Biology.   
 Instituto de Biologia Molecular de Barcelona
